# Supporting the wellbeing of caregivers of children on the autism spectrum: A qualitative report on experiences of attending group dance movement psychotherapy

**DOI:** 10.1371/journal.pone.0288626

**Published:** 2023-08-04

**Authors:** Supritha Aithal, Vicky Karkou, Stergios Makris, Themis Karaminis, Joanne Powell

**Affiliations:** 1 Research Centre for Arts and Wellbeing, Edge Hill University, Ormskirk, United Kingdom; 2 Faculty of Health, Social Care and Medicine, Edge Hill University, Ormskirk, United Kingdom; 3 Department of Psychology, Edge Hill University, Ormskirk, United Kingdom; University of Greenwich, UNITED KINGDOM

## Abstract

Caregivers of children on the autism spectrum can carry a significant amount of practical, psychological, and social demands and responsibilities that are highly stressful. A group Dance Movement Psychotherapy (DMP) was offered to facilitate the wellbeing of caregivers. In this article, we explore the experiences of the therapeutic processes and outcomes of the intervention from the perspectives of caregivers, the therapist, and the researcher/co-facilitator. **Method:** Four clusters of caregivers of children on the autism spectrum (N = 20 Mean age = 39.25 years) took part in five group DMP sessions lasting 90 minutes delivered across two special educational needs settings. Twenty reflective focus groups took place in total, with each taking place at the end of each DMP session. Participants were invited to capture their experiences through arts-based drawings, while therapist and participating researcher/co-facilitator kept session-based notes and arts-based reflections. These arts-based and verbal data were grouped to generate themes. **Results:** Six overarching themes emerged from the arts-based and verbal data with multiple subthemes that describe the contribution of DMP towards promoting caregivers’ wellbeing and identified key challenges in implementing the intervention. These themes are: (1) Beholding within and around; (2) Reflecting and reinforcing strengths; (3) Exchanging views; (4) Looking back and carrying forward; (5) Core benefits; and (6) Challenges to engage in DMP. **Conclusion:** Caregivers talked about their experience of participating in the DMP groups as positive and acknowledged the helpful and challenging aspects of taking part in DMP intervention. They appreciated the creative and expressive nature of the intervention to promote their emotional and social wellbeing. The challenges identified in the study indicate that further awareness is needed within school environments about the contribution arts therapies can make towards establishing appropriate and sustainable interventions for caregivers.

## Introduction

Around the world, one in every 100 children is identified with an autism spectrum disorder (ASD) presenting with varying degrees of health and social care needs [1]. The World Health Organization (WHO) [2] recognizes that people with ASD have complex needs on the one hand but the services available are inadequate. Despite several actions towards comprehensive and coordinated efforts for the management of ASD [3], accessibility and sustainability of appropriate services remains limited. As a result, it is often the case that caregivers carry significant responsibility as key support providers to children with ASD that can impact on their mental health. The term caregivers is used very broadly in this article to describe any person close to children with ASD and are in their primary circle to support their needs (e.g. parents and teachers).

Raising children with ASD is frequently associated with higher stress levels and reduced wellbeing when compared to the public and to caregivers of children with other disabilities [4]. However, caregivers’ needs are often neglected as intervention programmes generally focused on the children’s needs. Explanations for the stress experienced by caregivers can broadly be categorised under the following factors: child-related factors, family-related factors, socio-cultural factors, and political factors, each of which is discussed below.

### Child-related factors

The impact of some of the relevant child-related characteristics may mediate as stressors in caregivers of children with ASD. Child variables, such as the child’s age [5], their cognitive or developmental level [6], may act as some of the stressors [4,7,8]. It has been noted that the presence of emotional and behavioural problems amongst children considerably predicts the caregiver’s distress [4]. However, there have been contrasting debates on the relationship between the severity of ASD symptoms and general stress in caregivers [7,9] possibly due to cross-cultural differences in parenting beliefs and value system. Irrespective of the lack of consensus around the association between child’s ASD severity and caregiver stress, Porter et al. [10] argue that all caregivers of children with ASD need support. Thus, the focus of this study is broad and inclusive of different categories of caregivers of children with ASD.

### Family-related factors

Earlier studies highlight factors such as gender discrimination, difficult family dynamics, disturbed family routines, strained family relationships, marital status/single parenting may have an impact on depression and parenting stress [11,12]. Parenting stress has also found to have strong associations with marital discord, and child neglect and abuse [13]. When functioning well, family members, such as the partner/spouse, immediate or extended family members, can be a positive experience and act as a resource and support. Practical contributions towards childcare, household chores, and financial backing as well as emotional support from family can enable parents to cope with the challenges that caring for a child brings.

For teachers, it was found that salient factors especially relationship difficulties, marital status, gender, family bereavement, illness, family status and childcare issues tipped them over the edge [14]. It can be noted that Government reports [15,16] focus mainly on contextual factors: workload, the school’s circumstances, and the pupil’s behaviour in class. The Education Excellence Everywhere White Paper [16] fails to address other factors such as family commitments which can support the mental wellbeing of school staff. Ault et al. [17] identified that positive social support could play a key role in maintaining the wellbeing of caregivers. Therefore, this present study explored further ways to offer this support and the impact of creative group work.

### Socio-cultural factors

Three major interlinked factors that play a vital role in the parenting experience are the socio-economic status of the parents, their education level, and their social support system [18,19]. Regardless of the revenue, parents working in more lucrative and respected occupations were found to report stress more often than parents engaged in low-income occupations. Contrastingly, even though lesser financial constraints can enhance a parent’s ability to access limited available services (e.g., therapists or good schools) or adequate housing and may shield them from some of the damaging effects of stress providing a sense of control over one’s situation [20] they reported higher amount of stress. Families caring for children with ASD often experience social isolation due to the child’s need for sameness and routine, the child’s oversensitivity to sensory stimuli, or the child’s lack of adherence to social norms. Offering respite care, inclusivity, equal opportunities, cutting down tabooing and bullying of children are societal factors that can improve the quality of life of parents [19]. However, in reality, and for most parents, the environment remains difficult.

For teachers, several factors can contribute to a positive or negative work experience. For example, early stages of work experience, special educational background, role conflict, role ambiguity, and lack of administrative support can lead to burnout. It is not surprising that teachers may experience higher levels of burnout when they are entrusted with caring for children with ASD in addition to instructional and curriculum designing duties [21]. Research on stress in teachers (in general not specific to ASD) from Brown and Lan [22] as well as Glazzard and Rose [23] has identified some trigger points to these salient factors from the work environment and busy circumstances such as pressure of assessment and catering the academic expectations; the pressure of extracurricular activities; the unforeseen tasks; keeping up with the pace of change; and changes in school management staff. Positive relationships of teachers with children, co-workers, administrators, parents, other professionals, and experts can perform as buffers to counteract the risk of burnout [24]. Research also indicates a positive association between the long-term wellbeing of caregivers and their work environment in terms of happiness at school and job satisfaction as important shielding factors for burnout risk [25]. Thus, support from the school environment and resources provided by the administration may motivate the teachers to give their best in the classroom.

### Political factors

Political and the economic factors reflect on available funding, resources, research, and policies that have an impact on the health of children and caregivers of ASD. Having a child with ASD sometimes demands that one of the parents become a full-time caregiver [12]. If this happens, the family loses one source of income while at the same time paying expensive fees for specialised ASD interventions. Policies and associated funding packages are needed to reduce the impact of the financial hardships by making resources accessible along with adequate childcare services, child-minders, and psychotherapeutic support for caregivers. These all become crucial factors reducing exhaustion and influencing positively families’ wellbeing [26,27].

Similarly, Special Educational Needs (SEN) teachers require exceptional patience, dedication and skill, as well as internal and external resources, to meet the diverse abilities and increasingly varied behavioural, emotional and educational needs of children with ASD [28]. However, there is very little training available for them to address their own and the children’s emotional needs. Inadequate materialistic and non-materialistic rewards or career prospects (salary is linked to seniority) mean that professionals are not attracted to these roles unless they are driven by internal motivators. This leads to limited staff and few available resources to help cope with the high demands and requirements of such a SEN role [14]. This contrasts with the Teacher Education Theory that advocates that for teachers to be supported schools need to pay attention to both individual and contextual factors that contribute to staff wellbeing [29,30].

Overall, as a result of an interlay of the factors described above, the caregivers of children with ASD face more challenges than caregivers of children with other disabilities [9]. Parenting stress is also identified to mediate the relationship between child’s behavioural problems and decrease parenting self-efficacy [31]. Henceforth, the present study intends to explore creative ways of supporting caregivers of children with ASD.

### Interventions for caregivers

Most available interventions to support caregivers are mainly psychoeducational and focus on developing caregiving skills or raising awareness. A systematic review on interventions related to improving the quality of life of caregivers of children with ASD, synthesised the data from 21 studies between the years 2005–2016 [32]. Three different themes emerged from the types of interventions that were offered to the caregivers. The first theme was learning about ASD: interventions supported the caregiver to understand the diagnosis and ASD related information. In the next theme, interventions supported the caregivers on how to help and care for their child with ASD. The last theme focused on providing psychological care, networking opportunities, coaching, provision of resources for the caregivers. These approaches were commonly delivered in a group configuration and in collaboration with the family using distinct theoretical frameworks, for example the Model of Family Stress [33], the Family Partnership Model [34], and the Ecological Validity Framework [35]. Overall, the findings of this systematic review [32] show limited attention to improving the wellbeing of caregivers.

In recent years, family-centred approaches and interventions targeting school staff are increasing in arts therapies literature [36]. For instance, Pasiali [37] and Thompson [38] explored the effects of family-based music therapy on resilience, parental self-efficacy and supporting positive parenting practice. Dance Movement Psychotherapy (DMP), one of the arts therapies which uses movement or dance as form of creative expression has shown the potential to encourage clients to discover their innate strengths and resources toward achieving personal growth and recovery [39–41]. The study focusing on the parents of children with ASD was a short-term mixed-methods DMP intervention study that was conducted in India. Although the sample size was small, quantitative measures using Parenting Stress Index-SF and HAM-D showed promising results in favour of the DMP group regarding scores of stress and depression [42]. The same study [43] provided a theoretical framework to identify the process of resilience enhancement through DMP in parents of children with ASD. However, it was limited to only a small number of Indian mothers with a specific socio-cultural and economic background. Ultimately, there is a need to explore the transferability and generalisability to a larger group of caregivers, of different socio- cultural, economic, political, gender and other characteristics. Thus, the current study, which was conducted in the UK, aimed to capture multiple views and experiences (parents, teachers, therapists and co-facilitator/researcher) on the potential contribution of DMP towards the wellbeing of caregivers of children with ASD. In addition, new guidance by the Medical Research Council (MRC) [44] provides a framework for developing and evaluating complex interventions. This framework promotes the systematic development and evaluation of interventions. In line with this guidance, the present study was conducted to capture the helpful and unhelpful factors of the intervention from different perspectives with the research question:

*What are experiences of caregivers, therapist, and co-facilitator/researcher on DMP process and outcomes?*

## Methods

The data presented in this article is part of a larger doctoral study published by the first author, which included a mixed methods crossover research design involving children with ASD and their caregivers. Only the qualitative data that captured the lived experiences of the caregivers’ is presented here and this strand of the study was methodologically underpinned by Heidegger’s hermeneutic phenomenology [45]. At the time of the study, the first author had five years clinical experience of working as a speech language therapist and newly trained dance movement therapy practitioner with clients experiencing communication difficulties and their families in India. Inspiration for this project originated largely from her passion towards dance on the one hand and, on the other hand, while working with children on the autism spectrum she noticed that the caregivers were able to connect with their children better when they were relaxed. In addition, meaningful improvements in children were achievable when caregivers were less stressed. These anecdotal clinical observations of bi-directional relationship between children and caregivers eventually acted as the driving factor to explore the use of dance or movement to support the caregivers to support their children.

### Participants

Ethical approval for the study was granted by the Faculty of Arts and Sciences Research Ethics Committee of Edge Hill University. Purposive sampling was initiated by sending letters of invitation and project information sheets out to the headteachers of 18 different SEN settings in the North-West of England. Four schools expressed interest and permitted the researcher to mail invitation letters, participant information sheets and reply slips to the caregivers. Once approximately 15 eligible participants expressed interest from one location, participants were invited for providing detailed information about the intervention, research process and finally obtain their written informed consent if they wanted to take part in the study. A total of thirty-seven caregivers (including teachers, teaching assistants and parents) were recruited from two Special Educational Needs (SEN) school settings. Inclusion criteria were that they were caring for children aged 16 years or under with a diagnosed ASD. Data collection took place within a spacious room, confidential space with minimal disturbance, within the respective school premises between September 2018 to April 2019. Out of 37 participants, 20 belonged to the DMP intervention groups (N = 20), and it is this data which is presented in this qualitative report.

### Intervention

Caregivers were offered five DMP sessions lasting for 90 minutes each across a five–week period. Teachers and parents were grouped into four separate clusters with 5 participants in each for practical reasons and for maintaining professional boundaries. Attendance in each group varied each week. At first, following the template for intervention description and replication (TIDieR) guidelines [46], a DMP intervention protocol was developed, which is described in detail separately [47]. The protocol was designed to be integrative and eclectic in nature, and to be delivered flexibly based on the needs of the participants. Briefly the theoretical principles; overall process consisting of tools, techniques, and materials; and finally the session structure, which informed the DMP intervention were extracted from three projects [41–43,48] and incorporated into the protocol.

All sessions were delivered by an ADMP UK registered female dance movement psychotherapist and co-facilitated by the first author/female researcher; the latter of which was also responsible for the data collection and analysis. Sessions had a flexible structure with a new theme to explore each week. Each session started with a gentle opening ritual for a quick check-in followed by a warm-up involving mindful use of different body parts, improvised movement patterns, creating body rhythms, passing the leadership of the movements to different members of the group while the rest of the group echoed and mirrored the movements. The main themes that formed the core of each session included: knowing each other; identifying personal strengths and challenges; enjoyable moments with children; setting new vision and exploring new ways of being. The participants explored these themes creatively using creative movements and props such as fabrics, hoops, balloons, canopy scarves, large parachute, personal objects and sometimes music. Sessions ended with a closing ritual and reflections through words and creative medium. These verbal and creative reflections formed the source for the data presented in this study.

### Data collection

Data were collected in multiple forms and perspectives to answer the research question and enhance the trustworthiness of the data.

- Caregivers: Towards the end of every session, participants were invited to share their experiences through verbal and creative reflective focus groups. Open ended questions were asked on their experiences of the sessions and any changes they may have noticed in themselves and make a mark using visual artwork or creative movements. For example, what was this session all about for you? How do some of movement expressions/visual artwork relate to your life? What was the most enjoyable or challenging part of the session? Are there any movements or gestures of how you are feeling now? Artwork and movement experiences were considered as reference points to generate follow-up questions. The questions were piloted with co-researchers before the data collection.
- Therapist: The therapist was invited to offer verbal and movement reflections on the significant moments of the session after finishing every session and this was video recorded. The therapist also maintained written session notes.
- Researcher/co-facilitator: The first author actively took part in all the sessions as a participant researcher to gather data, offer technical support and to co-facilitate the sessions when necessary. Being observer-as-participant [49] created a platform for embodied recall of the experience during retrospective analysis of the video content [50]. Retrospective analysis of session video recordings were conducted to document the groups journey and participants movement qualities. Retrospective video observation allowed the researcher/co-facilitator to view the data from a distance.

### Analysis

The data obtained from four clusters of caregivers with five sessions for each cluster, gave the total data from twenty sessions with the total video footage of around 1800 minutes. The focus of the analysis revolved around exploring the views of the therapist, researcher and caregivers on the contribution of DMP towards their wellbeing. The contents were anonymised, transcribed and extracted to a spreadsheet to meet the research question to subject the content of these sessions to thematic analysis manually by the first author [49]. Contents of all the sessions were considered. However, priority was given to the key moments in the video sample which the therapist noted for every session. The process of ‘dialoguing’ was considered for the analysis of art-based data instead of interpreting the images from the researcher’s frame of reference [51]. Dialoguing was encouraged between the artists and images. After silently viewing the images or noticing their movements an aesthetic response between the art work and themselves was invited. For instance, the artists spoke what the use of space, colours, direction, intensity of lines, shapes etc meant to them with reference to their art work or movement. In the next phase the transcripts and annotated arts-based responses were collectively subjected to inductive thematic analysis for identifying, analysing and reporting patterns (themes) within data [52–54]. This process was intended to organize information, examine relationships, and identify trends in non-numerical and unstructured data [55].

Several measures that speak to qualitative and arts-based approaches have been used to establish the trustworthiness of this study. For qualitative and arts-based components of the study, credibility, transferability, dependability, and confirmability were considered [56]. The credibility aspects were monitored by implementing prolonged engagement and persistent observation with the participants and also with the data by the first author. Peer debriefing and discussion with supervisors also supported to enhance the credibility of the qualitative and arts-based analysis. The next aspect on transferability was addressed by paying attention to detailed description of the study’s settings, participants, procedures used to collect data and data analysis so that it helps other researchers assess whether the findings make sense in other similar contexts. In order to improve the dependability of the study, emphasis was given to the referential adequacy of the stored raw data and clarity in the description analysis, and interpretation to support step wise replication. Confirmability was achieved by taking measures such as paraphrasing and reflecting movements back to the participants to check if the researcher’s understanding of the materials shared by the participants reflected what they actually intended.

## Results

### Overview of the participants

As shown in Table 1, most of participants who took part in the intervention group were females (18/20) and their overall age range was between 28–56 years with a mean of 39.25 years. All the participants were British and the majority were from white ethnicity. Demographics on the marital status of the participants indicated that many of them were single parents raising more than one child. Their attendance to the sessions was poor, with only 50% of participants in the DMP intervention group attended at least 70% of the sessions until its end due to several personal and logistical challenges.

**Table 1:** Descriptive statistics of the participants background characteristics in the DMP intervention and standard care groups, separated by teacher/parent status.

Variables		DMP Intervention	
		Teachers	Parents
Centre	Location 1 (n)	4	5
	Location 2 (n)	5	6
Age (mean) and range		39.4 (32–56)	43.6 (36–51)
Gender (n) F- Female M-Male		9 F	9F, 2M
Ethnicity, (n)	White	9	9
	Black	-	2
	Asian and others	-	-
Marital Status (n)	Single parent	2	6
	Married	4	3
	Cohabiting	3	2
Number of children (median)		2	2
Number of children attending SEN (median)		-	2
Number of participants with at least 70% attendance		6	4

Verbal and non-verbal data from the participants, the observations from the therapist and the researcher who participated in the sessions were grouped together and it led to the emergence of six themes. These themes are:

1. Beholding within and around
2. Reinforcing and reflecting on strengths
3. Exchanging views
4. Looking back and carrying forward
5. Core benefits
6. Challenges to engage in DMP.

Most of the themes comprised of further subcategories. Table 1 offers an outline of the full codebook with all formulated themes and subthemes. Furthermore, the illustration depicts closely knitted links between the coded excerpts in each main and subcategory. Personal experiences shared by the caregivers are quoted with abbreviations such as P (number) for parents, T (number) for teachers, R (session number) for researcher and D (session number) for dance movement psychotherapist. Although, this is a qualitative analysis the excerpts were number coded, the frequency of occurrences of the themes and in which sessions were documented to enhance the specificity and trustworthiness of the data representation in this report. It is also believed that the information provided in Table 2 will be valuable to reveal and infer from the context in which such statements were made or when such observations were made.

**Table 2:** Qualitative themes codebook for caregivers.

Sl.no	Themes	Frequency of occurrence	Session Numbers (1–5)
1	**Beholding within and around** • *1*.*1Enhancing awareness and alertness* • *Unlocking the unfamiliar*	21	1, 3 and 5
2	**Reflecting and reinforcing strengths** • *Embracing Positivity* • *Recalling fun moments with children*	18	2 and 3
3	**Exchanging views** • *Structuring and compartmentalising* • *Sense of control* • *Escape* • *Confronting the challenges* • *Planning ahead* • *Acceptance*	19	3 and 4
4	**Looking back and carrying forward** • *Unprocessed baggage* • *Creative action plan*	16	1, 4 and 5
5	**Core benefits** • *Relaxation* • *Self-expression* • *Grounding and relishing the present* • *Letting go*	23	1, 4 and 5
6	**Challenges to engage in DMP** • *Contemplation about the approach* • *Readiness to trust and be playful* • *Access to emotional and symbolic content* • *Therapeutically safe environment*	14	1, 2, 3 and 4

**Fig 1:**
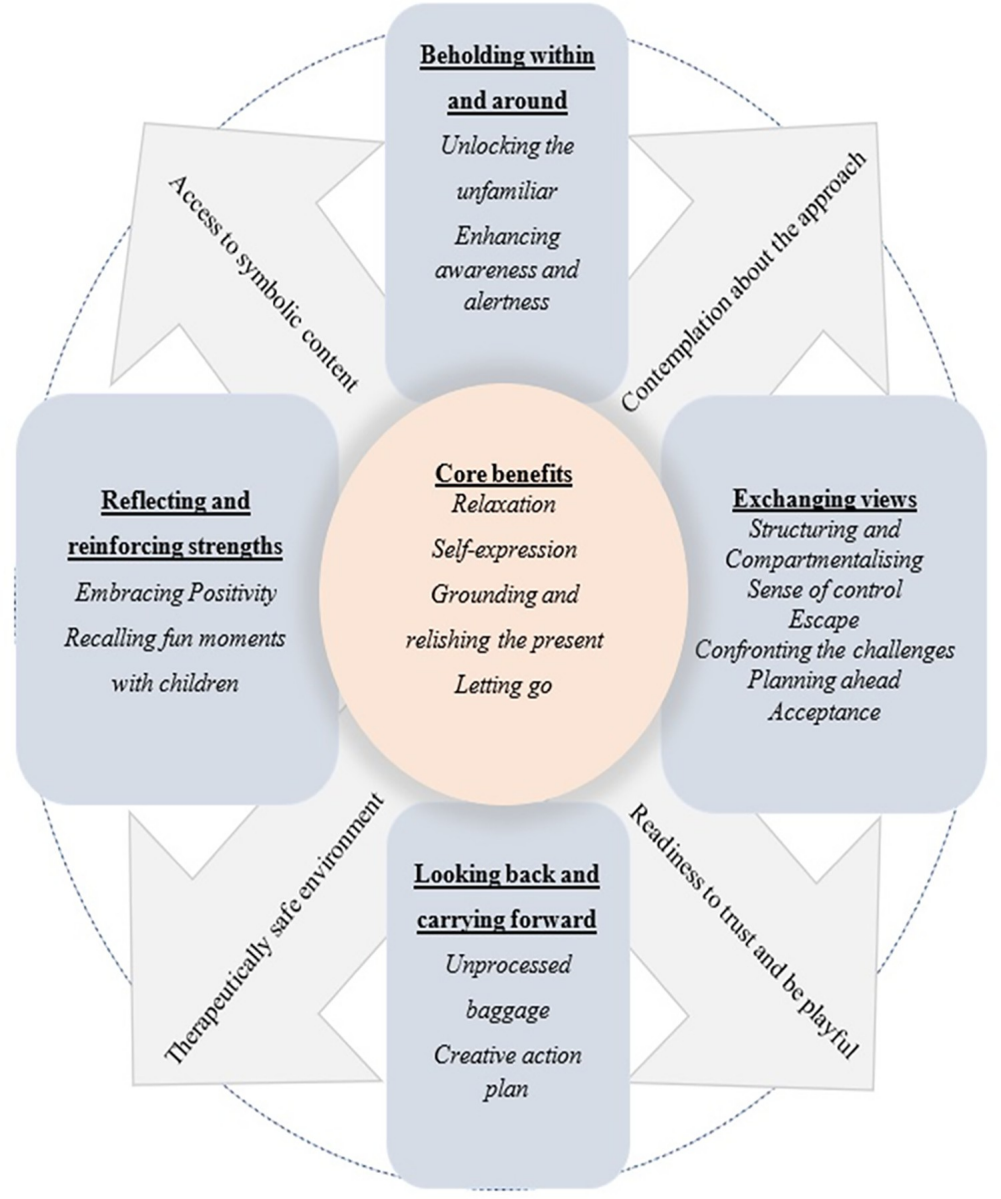
Summary of the qualitative results of caregivers.

The above six main themes and 20 sub-themes are illustrated as a three-layered process (Fig 1). The central part of the figure represents the core benefits from DMP as perceived by the group participants. The boxes surrounding the core benefits are helpful factors relating to the experience of the DMP process. Finally, the four arrow marks pointing outwards are the unhelpful factors that obstructed to engage in the therapeutic process.

### Beholding within and around

Many participants acknowledged during DMP sessions that the process of therapy encouraged them to observe themselves and their surroundings. They perceived that the sessions offered an opportunity to observe movement patterns of other participants in the group as well as sensations in their own bodies. These experiences were categorised under the following two themes:

#### Enhancing awareness and alertness

**Fig 2:**
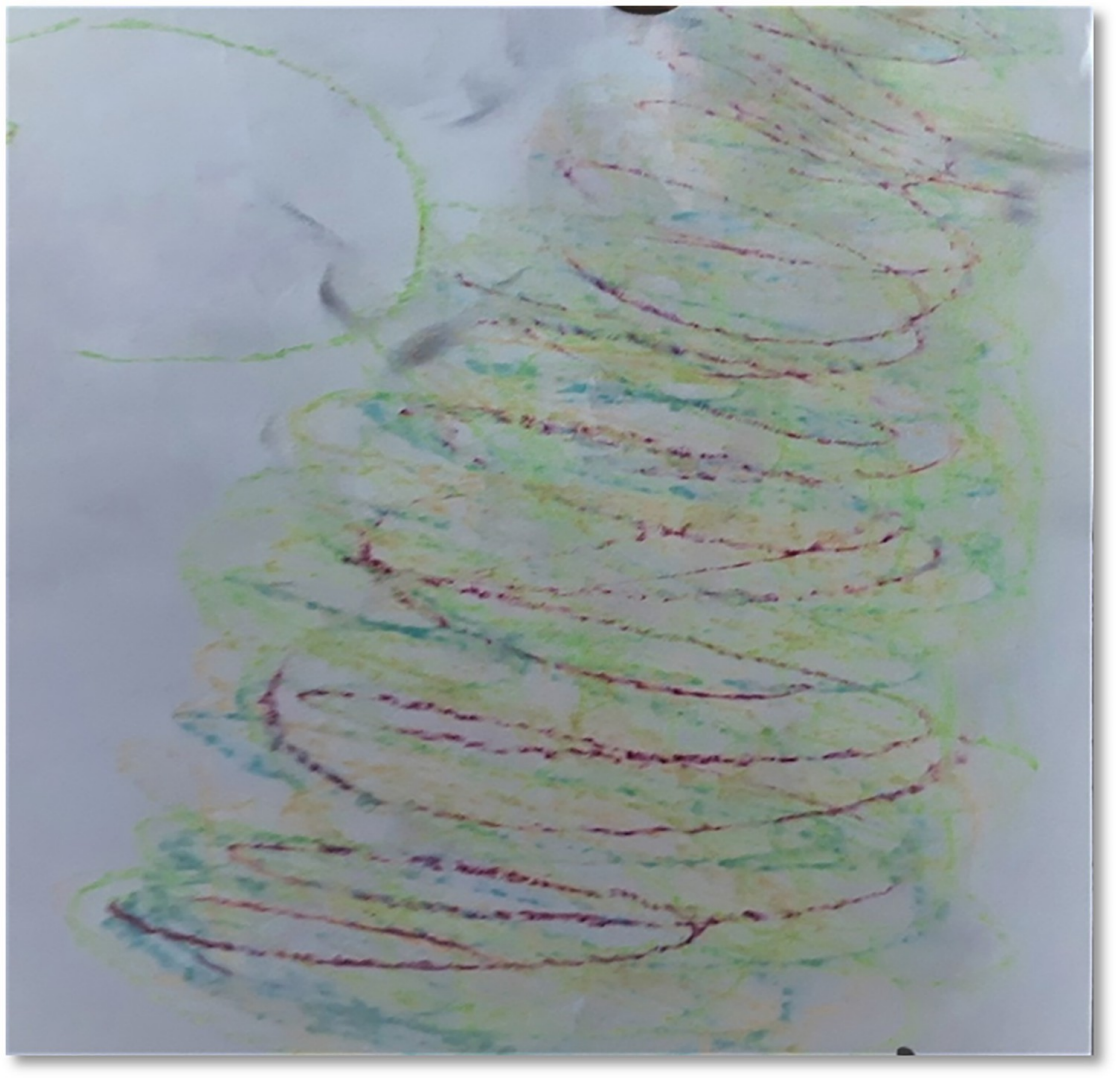
The rollercoaster.

As an opening ritual all the sessions began with checking in to notice feelings, physical sensations, aches, reactions, behaviours, and thoughts at that moment. One of the most frequent answers given by the participants when asked about body parts seeking attention were neck and shoulders. They reported *‘stiffness’*, *‘heaviness’*, *‘tightness’ and ‘problems’* in their neck, shoulders, and some joints (P2, P3, P4, T2, T3, T5, T6). This was not reported just once or twice but was repeated several times by both parents as well as teachers. In one of the sessions a participant reflected about the observations on her life in general based on the drawing that she created after moving *“I have realised how my life is in general*. *Rollercoaster like*, *with ups and downs*. *Powerful up swings and powerful down swings”* (P5, see Fig 2).

The therapist observed that the movements of the participants, especially of the parents, were restricted to the movements of body extremities with limited movements of upper torso. In addition, their movements were observed to be performed within a small kinesphere and with hesitancy. The researcher/co-facilitator noted ‘*fisted hands’*, *‘stiff wooden log-like postures’* during initial phases of the sessions (R1). When the therapist offered the participants some of these observations, a participant spoke about how DMP enhanced her alertness about herself and surroundings *“I think it makes you think a little bit more*. *It is helping my mind in thinking different postures mean different things*. *I am looking around and seeing how everybody has stood and sat*. *It tells us a lot by the posture regarding body image*, *feelings*, *emotions etc”* (P2).

#### Unlocking the unfamiliar

During these sessions, the participants shared their experiences on exploring unknown material and allowing to surprise themselves with the unfamiliar things that emerged during their movement explorations. For example, while reflecting on their movement experience, one of the participants shared the pleasure of engaging with the unfamiliar:

“*I don’t know*. *My hands were just free flowing (with a shrug)*. *I think we all start with something that we don’t know to do with and then we slowly start shaping it into something functional*. *Just feels good*. *I started somewhere*, *and from somewhere came this life you know*, *life with some kind of plant life*” *(T6)*.

Another participant indicated that she “*had surprisingly kind of comfortable positive experience” (T1)* when she was involved in a movement exploration activity.

**Fig 3:**
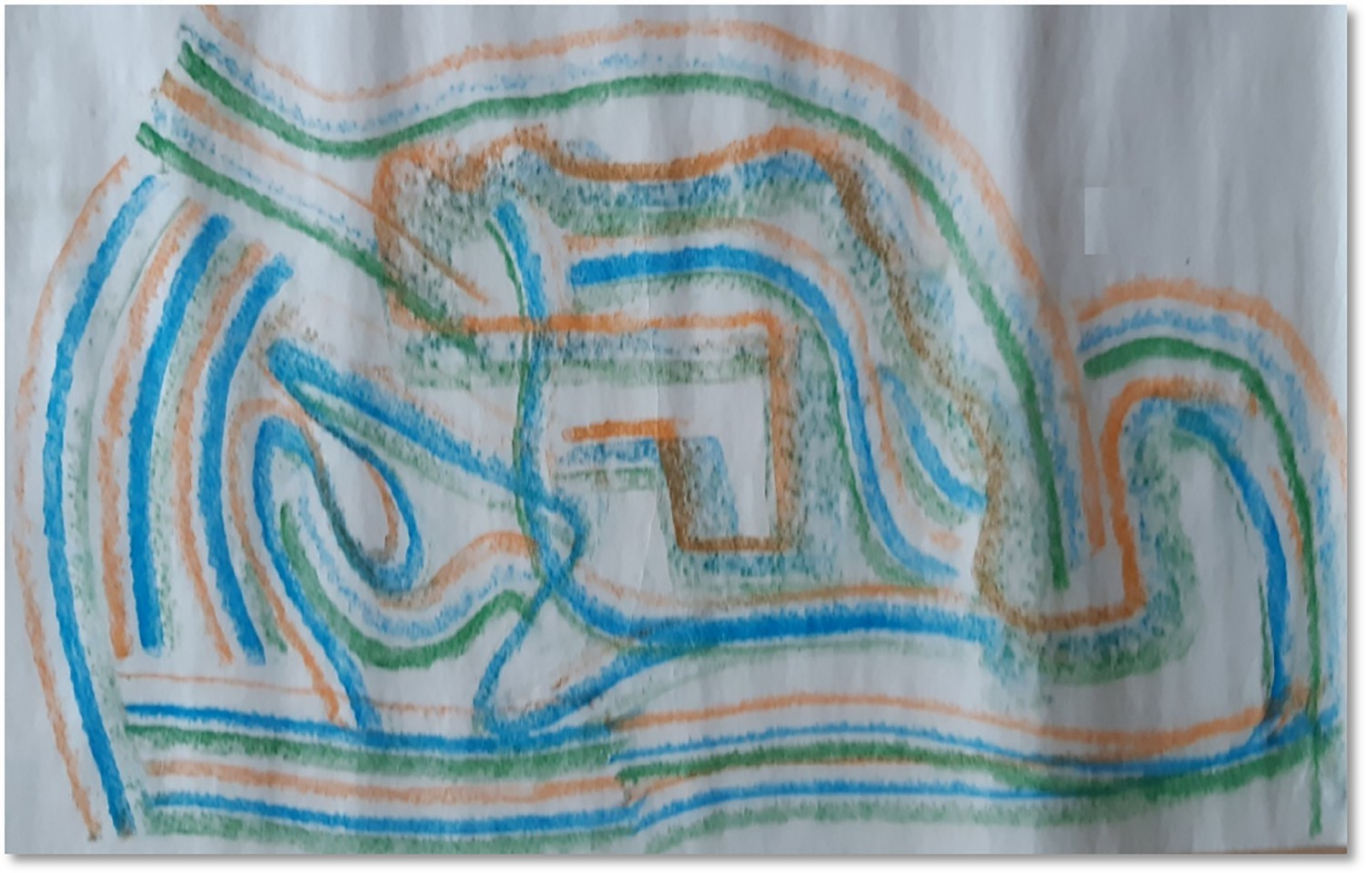
The unfamiliar space.

Some of the participants suggested that dealing with uncertainty and dwelling in an unfamiliar space within were challenging: “*In the beginning it is quite difficult as you don’t know what to expect*. *So there is a little bit of shyness and just not knowing what to do*. *A little bit wobbly*, *not too sure*, *one foot in*, *one foot out”* (T5, see Fig 3).

However, it was further reported that by allowing themselves to experiment with different movements arose novel strategies to deal with stressful events: *“I think we are adding new things to it and experimenting with actually if it is working well or not*. *hmm*.. *it will be flower or weed*. *It will need some pulling up at some point when it starts growing up” (T3)*.

### Reflecting and reinforcing strengths

Participants recognised and acknowledged that the strengths-based intervention approach used in the study was supportive. Some of the participants stated that the sessions provided a platform to identify some of the strengths within them. The therapist pointed out that the usual tendency of the participants was to talk about the successes they have had, but they did not want to attribute their own strengths to it. Henceforth, the therapist highlighted her role in identifying strengths in the participants and deliberately paraphrase their narrations to showcase the strengths in what they expressed.

“*And so I had to tell them this is because of your so and so quality or what you have done is with your strengths and resources*. *It is not a common practice to talk about strengths*. *People feel uncomfortable boasting about themselves*. *But it is helpful to have them reflected back to them*. *I guess it is what it felt like with the paper activity as well*” *(D3)*.

The therapist witnessed, reinforced, and validated their strengths. In one instance the therapist articulated *“It sounds like you have got a wealth of experience*. *Through dedication and consistency you know how to actually make all those threads happen”(D4)*. After this, a teacher (T2) acknowledged her years of experience as a resource and also credited her “*internal skills”* and said *“it takes a long time”* to mindfully inculcate them. Patience, creativity, persistence, loving, caring, reliable and good time management skills were some of the other qualities that were recognised as their strengths.

Subsequently, two sub-themes related to this main idea were identified in the data and they are described below.

#### Embracing positivity

The participants were encouraged to creatively explore some of the life events with the help of movements and props from a positive lens. They reported experiencing a sense of content and pleasure when looking back at something they had accomplished. One of the participants described *“It is just that the being positive like this has got a lot of benefits*. *I have been thinking about*, *the moments of success and the kind of response I have got and all that*. *Actually we have looked at lots of strengths and things that have gone well*. *And it’s been like very self-positive*. *It’s been really good and I am thinking like hmm*. *I want to lie down (T7)”*.

Some of the parents were also able to highlight the positive side of challenging circumstances of caring for children with ASD. One of the participants towards the end of the third session, drew her little family using bright colours and reflected saying *“I think the children that I have*, *make me more patient*. *It might sound quite strange here*. *But it is making me a better person*. *We are special” (P2)*.

The therapist while reflecting on one of the sequences in session 3 has reported on how the positive aspects expressed by the participants created an impression in her body and mind: ***“****Some of the proud moments expressed by T2 using a golden garment was in between the bubbles blown by T4*. *The interaction looked powerful*, *delightful or transcending*. *Sounds produced by T3 using a toy made me feel a positive drive emerging from my core” (D 3)*.

#### Recalling fun moments with children

There were instances in the sessions where the participants shared with the group some of the cheerful experiences with children. They reported that recalling special events and their special bonding with their children uplifted their moods and energy. The participants appreciated the opportunity to build a resource bank by rejoicing, recreating, embodying and being witnessed when they shared some of the joyful moments with their children. Some of the participants used abstract movements while some of them projected their feelings on to the props to express. For example, a parent expressed how happy she felt when she heard about her son’s work at school by gently lifting a balloon high above her head. She said, *“I loved that feeling up high” (P4)*. Another moment of playing with her son was expressed by reaching the balloon high up by supporting with her fingertips and letting it fall down into her cradling arms and hugging the balloon. Overall, as a common pattern it was noticed that the participants appeared confident while they were performing these movements as their postures lengthened in the vertical plane and they used broad movements with an expanded kinesphere. One of the teachers shared that she felt *“unbreakable”* and compared herself to *“a brick wall standing strong” (T6)*.

### Exchanging views

Over the period of DMP sessions while the participants shared their experiences and exchanged their views with the group, they appeared to be learning from each other. Especially in session 3 and 4 the participants after engaging movement explorations, described different coping styles. In addition, they discussed several child management techniques that worked with their children and some of which were acknowledged by the members of the group as useful. They stated that they would consider some of them as they believed some of the strategies were worth implementing in their personal life and with their children. Sometimes the participants just acknowledged the individual differences and processed why their personal preferences differed from others or why such an approach would not be applicable to them. Different patterns that were noticed overall are categorised under the following sub-themes:

#### Structuring and compartmentalising

**Fig 4:**
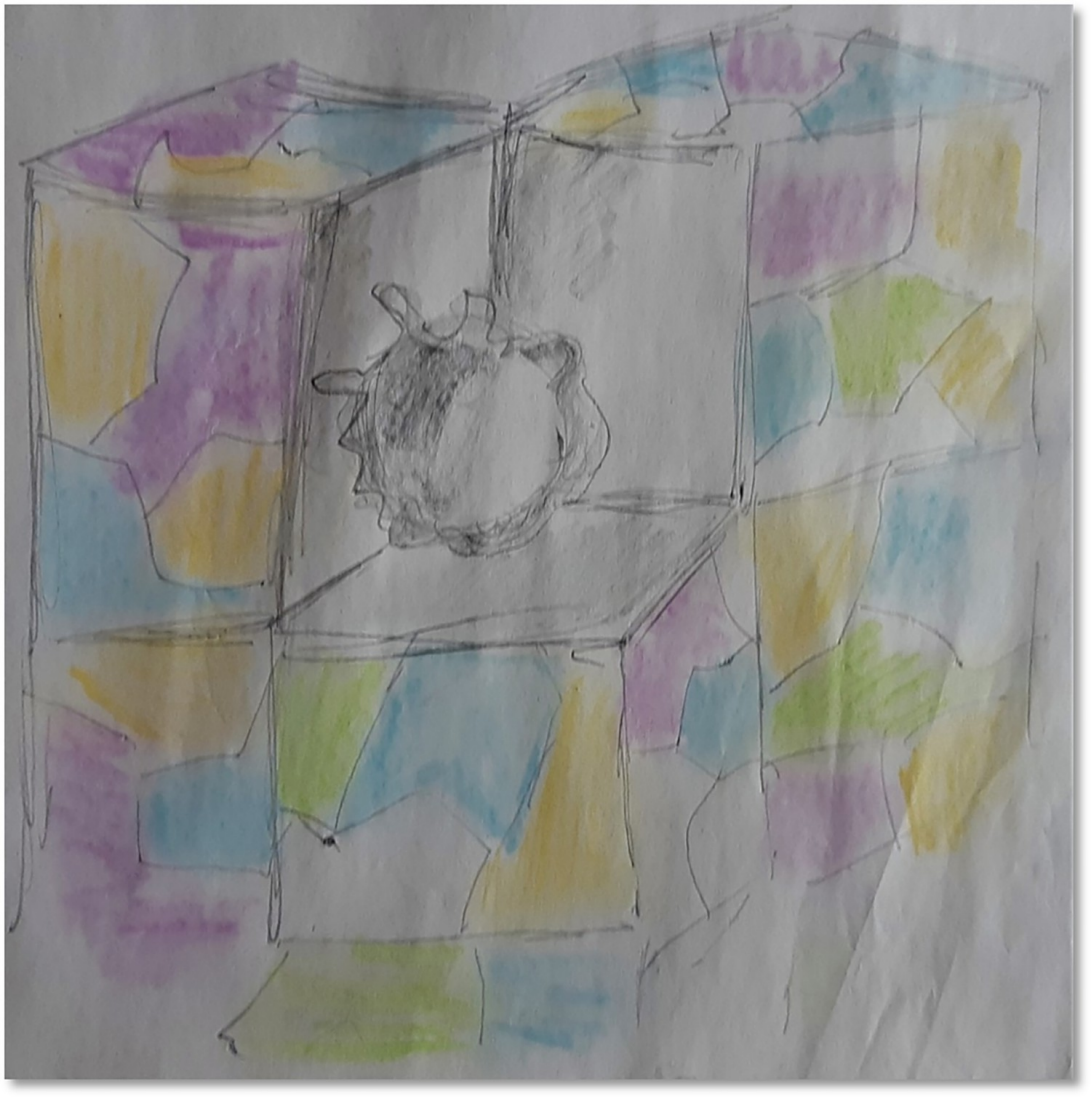
Structuring and compartmentalising.

Participants reported that some of the DMP activities facilitated them to know how they had compartmentalised resources and challenges. They realised that tidying them away at regular intervals was necessary to maintain wellbeing. Knowing the ways through which participants can have strategies to release challenges and sometimes keep them without being affected by those challenges were discussed. *“Trying to do some organising and tiding up really*. *I was thinking about what you said about challenges before*. *I actually realised that there is a space in my mind for challenges*, *but it just has to sit in the right space and make sure that I don’t over think that it is challenging and giving it right support*. *It is not locked in*. *It is free to go if it wants to go”* (T4, see Fig 4).

#### Escape

Another pattern that was noticed during the discussion on dealing with challenges was avoiding or escaping from acknowledging the challenges. One of the participants expressed her choice to move away from undesirable and negative events like water off a duck’s back as her intrinsic style. Her revelation during one of the activities during session 4 highlighted that there is a need for some sort of balance in the significance and weightage that is given for resources and challenges. She voiced *“When I was doing the obstacle course it was very negative*. *I saw this (glittery ball) on the table*. *I had a little bit of play with it*. *It reminded me that I had to be happy*. *Something for me is our own negative thing is problem to something*. *So*, *I just need to be happy*. *It is that simple*. *Balance it all” (T5)*.

#### Sense of control

**Fig 5:**
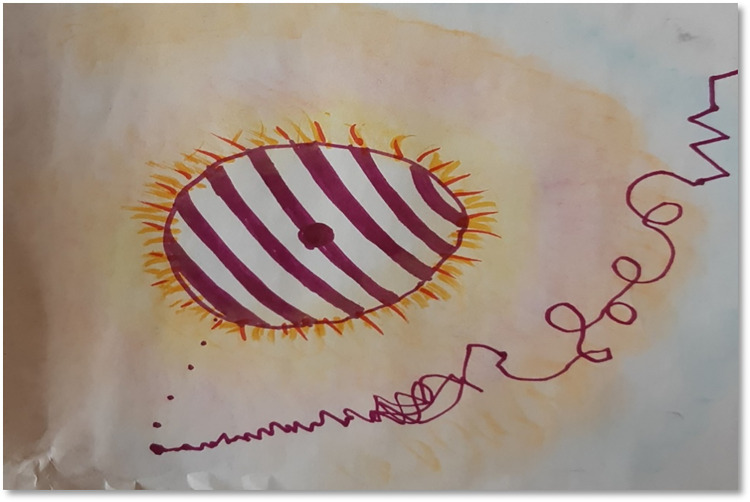
Sense of control.

One of the participants after a movement improvisation activity with a balloon filled with their challenges session described how the strategy to deal with her challenge unravelled. She expressed that she realised that she must be more specific to identify challenges, have some control over them instead of just letting them overwhelm her. Pointing to the balloon she revealed *“This is the challenge*. *But that is okay*. *It’s there*. *I still need to do all the other things*. *Just having a side to an eye*. *Just being in control of them*. *Once all the challenges were in here (balloon) I was able to move it in a way I wanted to and it was not moving me*. *That sense of control over managing the challenges is something that I would want to have”* (P3, See Fig 5).

#### Confronting challenges

The participants also discovered and shared various ways that required modifications or slight reworkings of their environment to turn around the challenging pathways into barrier free space. Some of them expressed that after shifting around certain objects in the obstacle path that they had created; it did not remain as an obstacle path anymore. A couple of participants said that a modification with addition or deletion of some movements provided a whole new perspective to the so-called challenge that had believed to be a challenge until then. For instance, a participant shared that she added *“a pirouette and quick spin around to catch up with everything and adopt to different things helped me carry on forward” (P4)*.

#### Planning ahead

Some of the participants preferred to plan to face the challenges that they anticipated. It was illustrated as a multi-layered approach by one of the participants where she had several layers of colours with many dots. *“the little blue dots and the challenges that are up against in the next six months*, *it is gruelling*. *I have to move some of these layers to allow for the difficulties to be dealt with the opportunities at that time*. *A radii kind of movement from big fish to little fish*. *I got to move things in between things here and there*. *I will get there in the end but I’ll have got a few obstacles to cross first” (T1)*.

#### Acceptance

One of the views most expressed and agreed by all the participants was accepting the circumstances they were in. Everyone (parents and teachers) agreed that there were some aspects which were beyond control and the challenges had to be accepted as they were. One of the participants described a situation which was very common in a classroom setting *“In my class there is a lot of energy*. *I was thinking about a specifically particularly a stressful day when one of my TA was off*. *I was literally*, *every time I sat down a student would call and then had to get up and move back and forth*. *Every time I took a step forward I had to back again to sort things out*. *Sort someone out sort someone out*.. *so this is kind of a lot of different colours and energy and they all need different things and I’m trying to fix it all which is not possible” (T2)*. She further described movement exploration of this situation in this way *“At one point I was looking at the tennis ball and I was thinking you (the ball) come to me*, *I’m not coming over there again*. *But then it’s not gonna come over to me*. *I’ll have to get up and go and get it*. *When it starts moving I feel like stop*, *stop it*!! *alright I’ll come over and stop” (T2)*.

**Fig 6:**
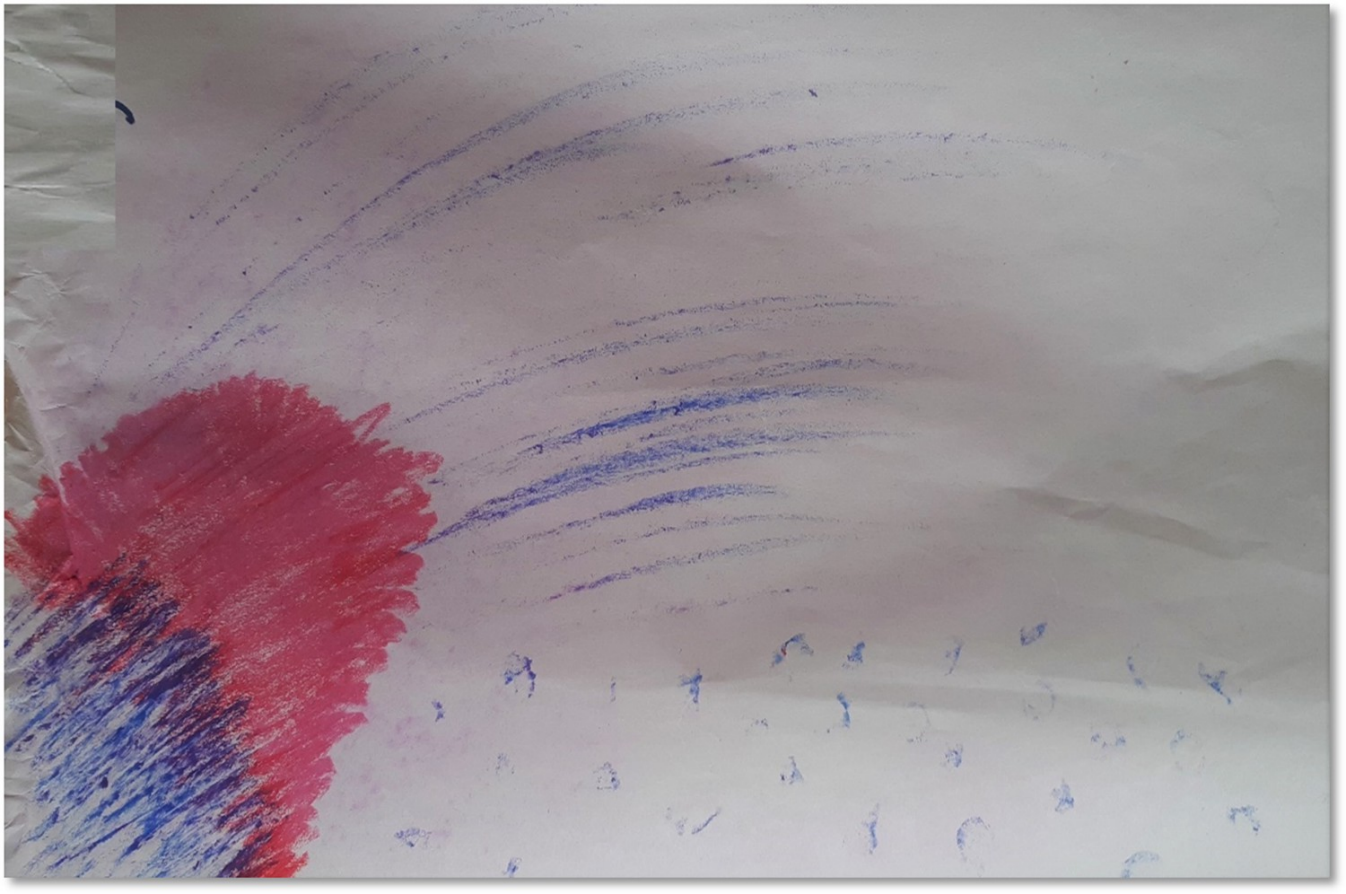
Sweeping the mess.

Another participant reiterated a similar circumstance and highlighted that acceptance of things as they are is sometimes the only option and becomes important to enjoy even when the situation is chaotic and out of control. She uttered *“I’m having my fun thing here*. *That’s my vision*. *I decided go with the flow*. *So that’s why I just let my balloon just go off*. *Sometimes you will just have to let go the way it is*. *I was tiding up a little bit when I was doing my obstacle course for a while but later decided to let it go*. *Okay*.. *let the chaos come*. *It’s gonna come all the way just let it go with it anyway*. *Fighting it is gonna be more of a challenge” (P4)*. Likewise, the theme of acceptance unearthed in an artwork which was created in response to the movement exploration: “*I know when I saw all the dust on*, *it was getting quite messy*. *I was trying to sort that out by sweeping it all*. *But still you can see those little dust particles on the paper*. *I guess it is always going to be there”* (T6, See Fig 6).

### Looking back and carrying forward

It was evident in some of the participants’ art and movement-based expressions that they were carrying unprocessed materials from their past experiences. The participants have referred to their childhood memories as well as tragedies of their recent past while creatively engaging with the arts. In addition, worries about the future of their children and eagerness to learn arts-based skills to implement with the children were noticed. These two patterns where the participants oscillated backwards and forwards in their thoughts are discussed separately in the following sections.

#### Unprocessed baggage

**Fig 7:**
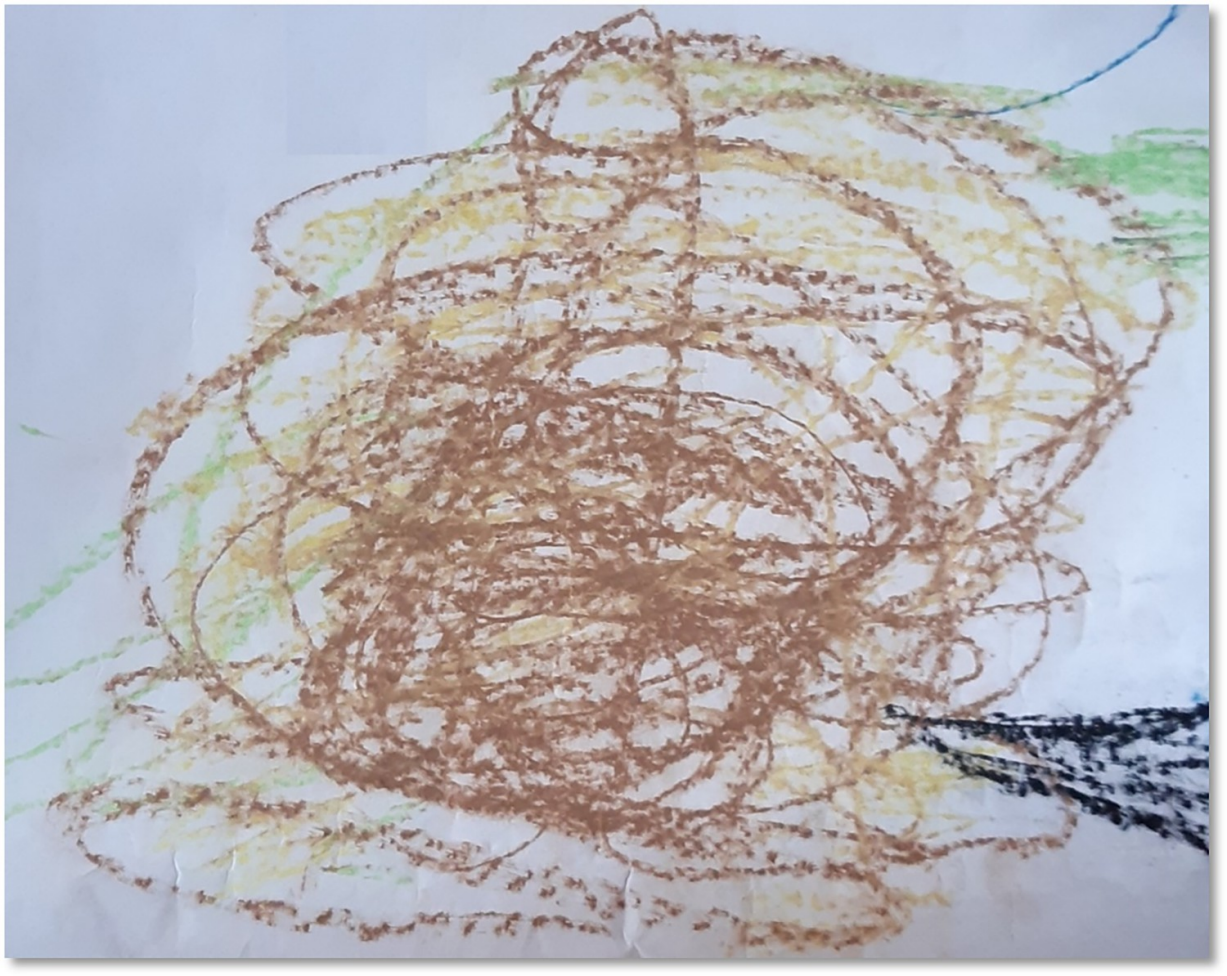
Just some colours and lines.

During movement-based and verbal interactions with the participants, it was noticeable that they were carrying psychological baggage of a lifetime on their shoulders. Sometimes this extra burden they carried reflected on as physical symptoms and had an impact on their present. Although attempts were made to encourage the participants to acknowledge and process those issues, not many of them were in fact ready to face and address the issues. Sometimes they appeared to be ignorant about the unprocessed psychological baggage and at times it seemed they were not ready or equipped to process the emerging materials. For instance, P1 during movement exploration had series of movements where he walked backwards with backhand strokes and created a brown and yellow tangled pile in response to his movement experience. When inquired about what that meant to him, he replied “*nothing*, *just some colours and lines”* (P1, See Fig 7). The therapist interpreted that image as communication of his untreated inner conflicts or confusions filled with disdain and disgust. However, the participant ducked further processing and reported as something insignificant. There were several such instances across the four clusters. One of the participants recalled childhood days as the memory was evoked by a hand gesture using two hands setting the fingers apart. It was only one participant who ultimately opened up in the last session about her unhealed grief of losing her father. Since it was the final session, there was not much time to process it further.

#### Creative action plan

The participants expressed their interests to take away their learnings from the session outside the therapeutic setting and implement in their actual life. The meanings and personal discoveries were creatively converted as action plans to be able implement practically. Some of the points were short-term goals and had an immediate impact. For instance, one of the participants revealed that she would implement some of the coping strategies that she realised from session 4 in the commencing week: “*I have got a few projects going on which we don’t know where this will go completely*. *But*, *well definitely selectively prioritising tasks will be useful” (T3)*.

There were also occasions where the personal discoveries had long-term impact. A participant expressed *“I don’t think it is something that has immediate effect*. *But*, *I know over the course of time I have to be prepared*, *move some ideas to face it in a different way than I would normally” (P2)*.

The therapist also validated their choices and encouraged them to carry their decisions forward: *“It looks like you have got your own back now*. *Literally that hand movement is just like I have had this meeting of myself and I have got my own back for going forwards over the next week” (D3)*. These self-imposed plans were reviewed in the beginning of every session while the participants checked-in to the sessions. In addition, the participants also reported that they left the session being hopeful about the future.

### Core benefits

The participants expressed different ways in which DMP had an impact on their body and mind. These were the immediate changes that they perceived within themselves by comparing their movements at the beginning and towards the end of the sessions. The caregivers noticed that they were able to experience being relaxed, express themselves, feel grounded and enjoy the moment by letting go unnecessary thoughts and stressors from their mind and body.

#### Relaxation

**Fig 8:**
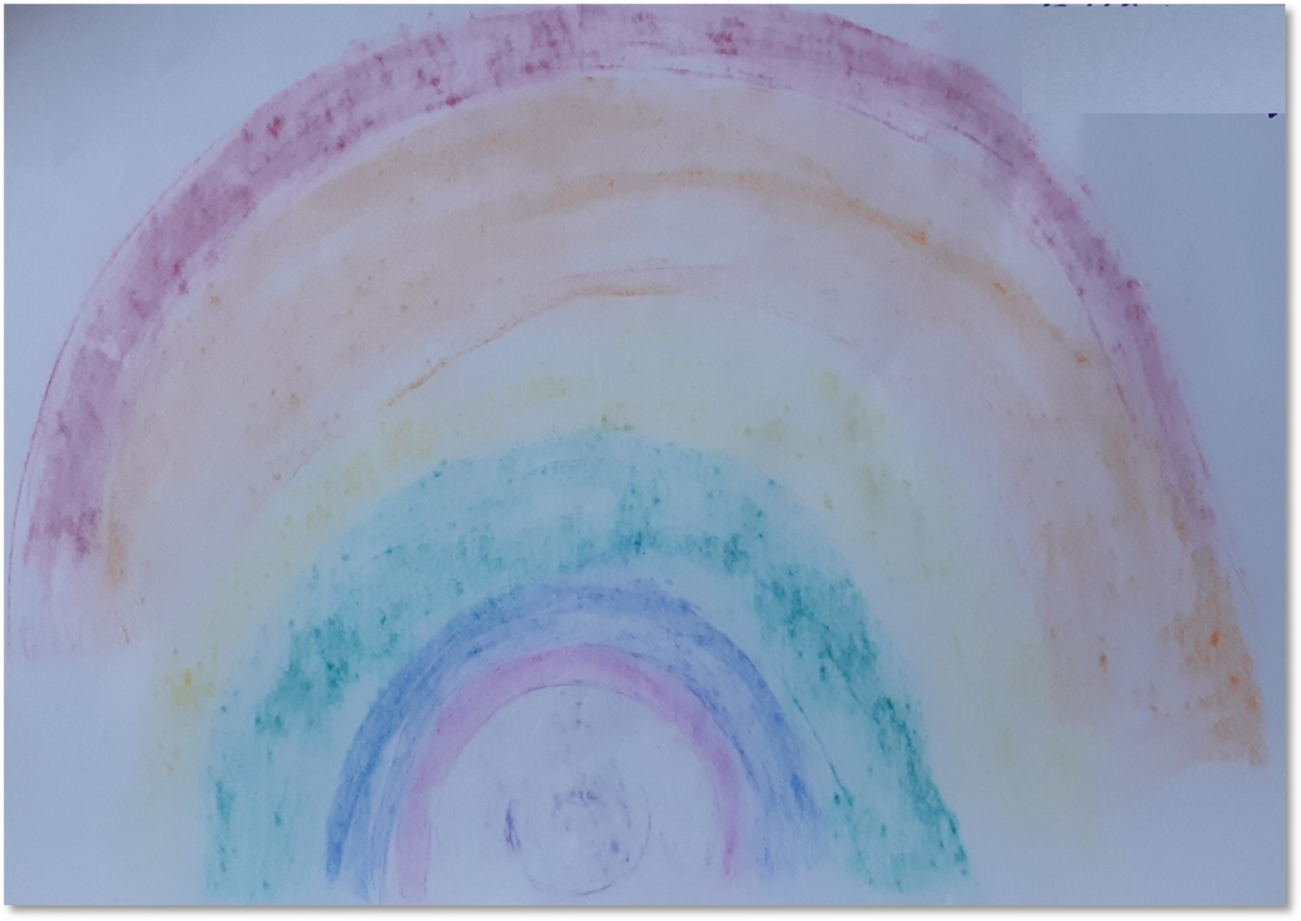
Bringing calmness.

This was one of the most frequently used words by all the participants. They perceived these DMP sessions as a platform to unwind and bring clarity in their thought processes. Some of them reported a calmer composure of mind, low tension, and anxiety. The participants also compared the DMP process to Yoga from their earlier experiences. It was also conveyed that the opportunities to stretch and loosen the body and focus on breath facilitated them to relax. One of the participants placed several layers of garment over her head and represented that they were all the worries sitting there in her head where there was no scope for anything to fully pass through it. She described her experience in DMP sessions by unwinding different layers of the garment like the layers of an onion. Another participant described that her experience was *“like water from the top rolling down the shoulders and now I’m relaxed” (P4)*. Similarly, another participant dropped her arms and mentioned that she was able to slacken the tension around her shoulders after session 4. The participants’ movements were observed to be using light and free flowing body efforts while describing their relaxed sense of being. In addition, the relaxed state of mind reflected on their choice of colours during closing self-reflection using artwork. A caregiver stated: *“I started off with purple*, *because it is the colour I like*. *It brings calmness to me”* (T3, See Fig 8).

#### Self-expression

**Fig 9:**
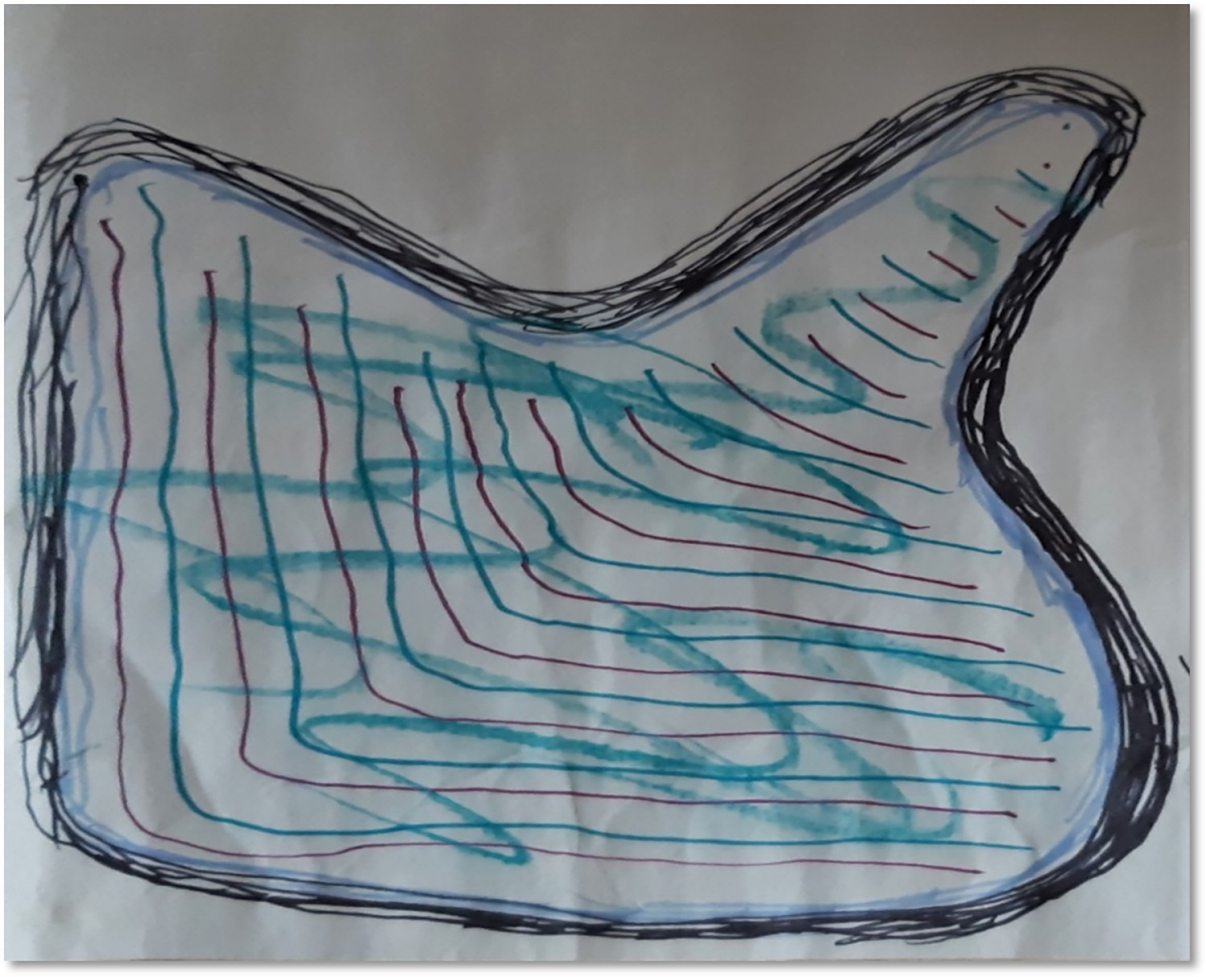
An organised chaos.

The participants noticed that DMP had given them an opportunity to be themselves by expressing their thoughts and feelings without any hindrance and fear of being judged. They mentioned that they were able to break out of their generalised and fixed patterns of living and experience some sort of change in their way of living. For instance, a parent expressed that *“routine and the straightness are the most challenging kind of aspects”* (P2, See Fig 9). She explored this challenge through movements and represented her experience through art work. She represented her son’s routines with straight lines and her desire to bend and take different curves around that. After movement exploration she decided to overwrite on the original drawing by adding some chaotic marks on the original straight lines. She reported that she had found alternate ways and some room for a curve or divergence in between straight lines. She specified that finding a break from routine was something that was not practically feasible. Although organised chaos within the set routine was something achievable by containing the chaos within a boundary. Another parent looking at her art work created towards the end of the session expressed that *“I have been carrying a lot of stuff while managing to maintain a positive outlook in the front whilst also dealing with the stuff inside or underneath*. *I can see it being stormed up in my drawing”*. The therapist observed that the participants had used the whole space when they had expressed them fully. Otherwise, they seemed stagnant and used very little space in the room. She described “*People have extended a bit further than what they would usually do*. *Felt it flowed well today” (D4)*.

#### Grounding and relishing the present

Reports from the participants emphasised that the sessions helped them to feel grounded and keep them active and involved in the present. They collectively approved that the sessions supported them to reorient to the here-and-now and in reality. It enhanced their alertness, centred energy, focus, slowed them down and helped in managing overwhelming or intense anxiety. For instance, a teacher described *“I think the time has passed really really fast*. *Before coming in its like I’ve got to do this that*, *what to do for the week*. *Once you are in you just let go” (T2)*. In the same way, a parent unlocked her knees, placed her palms on the ground and said *“I felt being connected to the floor” (P4)*. One more participant conveyed “*While I was walking my cheeks went up like I was smiling*. *Probably the music*. *I realised may be I was being fast and then reduced my swiftness”(T7)*.

#### Letting go

Caregivers who participated in the DMP sessions revealed that the process helped them to move out of their shell. They expressed that they had *“opened up” (P2*, *P3*, *T1*, *T2*, *T4)*. In fact, letting go of unnecessary thoughts, cleansing thoughts and perspectives had turned one of the clusters’ themes as rubbing or brushing body was the source for movement improvisation. The therapist exclaimed “*this whacking it out thing seemed to be like a group theme*. *How do we clean ourselves and get back into this (opened arms)” (D2)*. At the end of session 3 after creatively reconnoitring through movements on various challenges a participant described *“It was worthwhile*. *Kind of letting go*. *It was quite an experience with force coming from underneath (with pronounced exhalation)*. *I have burst the balloon*. *That’s all done now (with a smiley gesture)”(P2)*. She reported that she was content with her choice and that she was able to relieve herself from pressing and challenging feelings.

### Challenges to engage in DMP

Alongside the positive aspects of DMP, the interviews, remarks and reflective narrations made by the therapist and observations documented by the researcher strikingly revealed several challenges. There were several factors noticed in the participants that impeded their personal growth and group development. Across all four clusters a strong group bonding was not noticed. In fact, in some instances a group never formed, especially with the parents. Several unhelpful factors that hindered the therapeutic engagement were observed and they were categorised under following themes.

#### Contemplation about the approach

**Fig 10:**
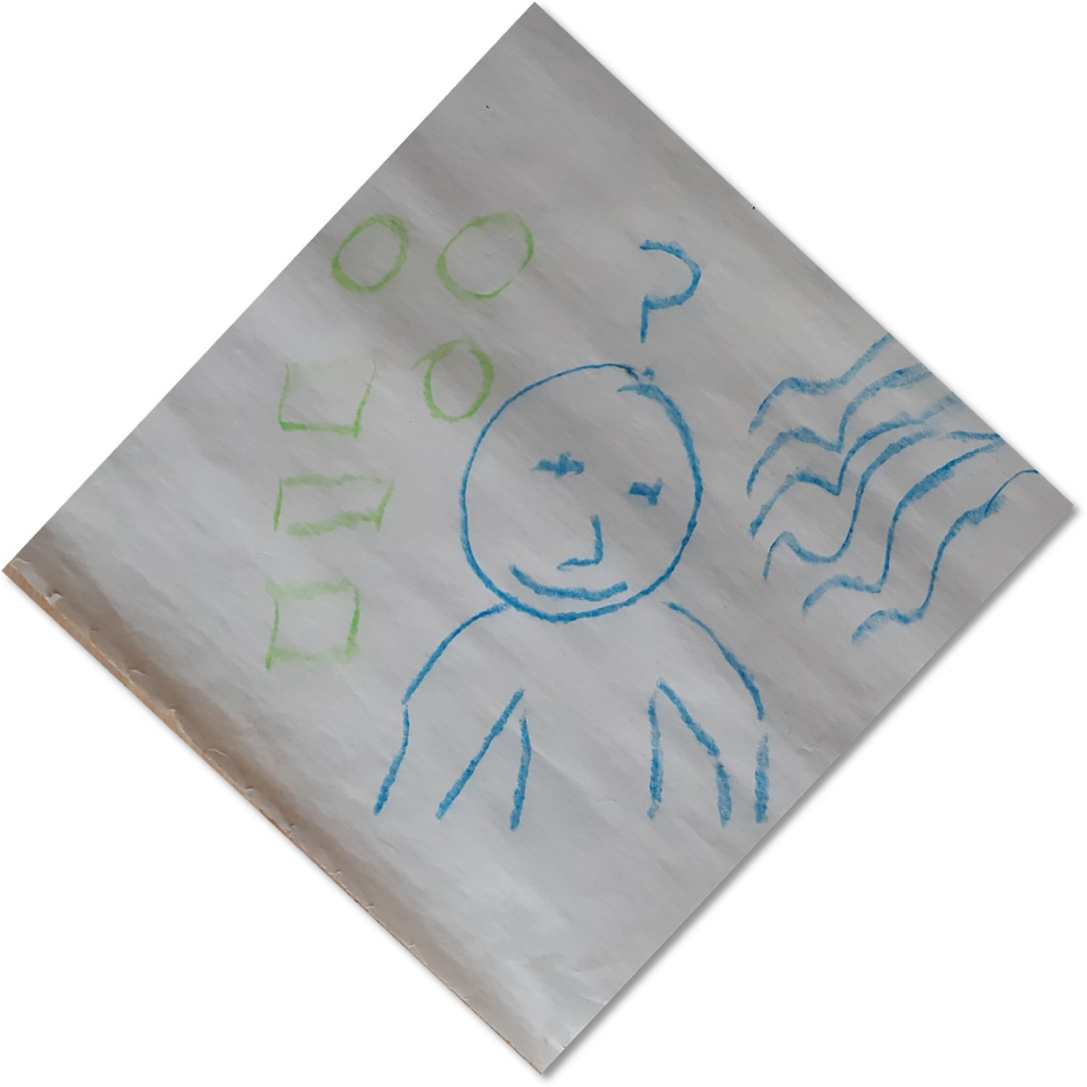
What is DMP really about?.

Preconceived attitudes, conceptual mis-associations, and expectations from DMP played a vital role in the responses and engagement of the participants. In terms of preconceived attitudes, many participants were expecting some sort of dance-based tool kit, skill set or series of steps that they could learn. Some of the participants were convinced that DMP is something for their personal wellbeing. Most of the times their focus was on how useful will it be for their children. The expectation was that it would be some type psychoeducational or behavioural training programme through which they could learn certain techniques which can be directly implemented in training or teaching children. After attending four sessions one parent mentioned “*I was expecting to learn teaching skills and strategies to manage my son’s energy*. *What you are doing is good*. *But*, *this is just not for me*. *Probably*, *I can see my son may benefit from this” (P1)*. There were only two male participants who had signed up for the programme and one of them said *“I was just curious and came in to see what this is all about really*. *I don’t think this is for lads”* (P7, See Fig 10). These were some of the responses received from the participants despite providing participant information sheet and verbal description about the approach provided before commencing the intervention programme.

#### Readiness to trust and be playful

The dynamics of the group was severely impacted by the willingness of the participants to step out of their comfort zone and engage playfully with the therapist’s invitations. Some of the participants expressed high resistance and anxiety to explore different movements and look inwardly. Their uneasiness was evident in their body language with crossed hands and tensed torso. Regardless of the measures taken to design the session inclusive and engaging to all genders and keep the tasks as simple as passing a ball, one of the participants stood near the wall resting one of his folded legs on to the wall and appeared to express embarrassment to pass the ball. The sessions were sometimes perceived as being *“silly” (P5)* and only some participants were able to let go of their adultness or maturity. Another factor which was noticed to have impacted on the group dynamics was varying degree of familiarity among the group members. Especially with the teachers working in the same place and the prior work relationships played an important role in the authenticity of the therapeutic process. There were occasions where the group members were going off rhythm and did not sync-in with the partners and sometimes the other extreme where the friends would pair up as an instinct during some of the activities.

#### Access to emotional and symbolic content

Some participants did not find it easy to engage with abstract, explorative and inward-looking activities. Their willingness to access emotions and attach symbolic meaning to movements and artwork and create connections with their lives seemed a challenge. Their emotional availability appeared insulated as the props were used in a concrete and literal fashion. For instance, when feathers were used as a prop they always remained as feathers. Their verbal reflections also appeared concrete as there were more outward references more than emotional or personal life references. Their movements were direct and bound. Having noticed these qualities, the therapist gave further thought about adjusting the work in such a way that everyone could respond in their own way “*Some people are more concrete thinkers*, *aren’t they*? *Especially T5*, *like you know the clock thing might gonna work for her*. *She kind of wants more boxes*, *contained concrete and mundane while the rest of us can float around everywhere”*.

#### Therapeutically safe environment

Even though the participants did not mention the therapeutic space, it was obvious that several factors were disruptive to the therapeutic process from therapist and researcher’s perspectives. An unsettling climate in the SEN school setting was not ideal for DMP. The therapist and the researcher reflected, discussed, and implemented several measures. In spite of all the efforts and precautionary steps there were numerous interruptions for the participants to engage in the session. For instance, regardless of the *do not disturb* notice on the door, teachers were suddenly called out for *“urgent meetings”*. The therapist reported that it disturbed the flow of her thought process and plans.

## Discussion

In our literature review we have extensively documented the challenges faced by the caregivers of children with ASD and how wellbeing could be negatively impacted due to stress and burnout that arise while caring for children with ASD [57]. This was no different in the participants of the current study where emotional and social wellbeing were at risk and was reflecting on their mental health. Hence, the focus was on how DMP could promote emotional and social wellbeing. In this qualitative strand, attention was given to the processes involved in DMP and the perceived outcomes of taking part in DMP journey. The findings show that the process instigated the participants to look at their self-schema, identify their strengths as well as their helpful and unhelpful coping styles. These processes might have facilitated the participants to experience some positive outcomes immediately during the sessions. Alongside these helpful factors the study identified several defensive and unhelpful factors which might have obstructed the process to reach deeper to potentially experience greater positive impacts of DMP.

The findings are consistent with the belief that DMP enhances body awareness and draws attention to sensations, feelings and thoughts which might have otherwise gone unnoticed [43,58,59]. As the theme beholding within and around indicates, the awareness was not just limited to developing an awareness of one’s self. During this group DMP, the participants also noticed every other body around them. It is possible that mindful movement-based activities such as creating body rhythms, passing the leadership to lead the movements while the rest of the group echoed and mirrored the movements might have encouraged the participants to scan their bodies and identify somatization [60] as an invisible injury due to stress and burnout which can only felt when listened to the body. The protocol upheld the principle of starting the process from the place where the participants are and working with the ‘now’ [59]. This could have been one of the reasons why participants of this study highlighted the value of relaxing body and mind.

Another theme that has emerged in this study is the value of emphasising strengths. As recommended by Miranda et al. [61] since ignoring the stressors faced by the caregivers may not be a feasible option, enhancing their coping abilities must be the focus of interventions for these participants. The results indicate that DMP has facilitated caregivers to turn stressful situations into finding positive meaning. This is in line with the theory that resilience is evident when individuals are able to use positive experiences and strengths to rebound from stressful encounters [62]. In DMP the movement materials and creative use of props can emerge as a form of non-verbal story-telling, a movement and embodied narrative of key moments. As mentioned in the results, identifying and talking about personal strengths did not come instinctively to the participants. Hence, the role of the therapist in kinaesthetically picking up the positive dimensions in the movement narration and reflecting it back might have been critical to support participants to find meaning and personal strengths in their creative expressions. This in turn could have led to experiencing greater positive self-regard [63]. It supports the view of positive psychology of the value of prioritising the enhancement of participants’ strengths and resources over dealing with the challenges [64].

Consistent with the results of the previous study conducted in India with the mothers of children with ASD [43], one of the themes in this study too revolved around exchanging views on different ways of coping and cognitive adaptation. It is possible that normalising and accepting the ups and downs in life are common experiences across groups and culture. Movement explorations might have facilitated some of the participants to unlock the paralysing loop of unwanted thoughts and take action to confront or embrace life’s challenges. At least while participating in DMP sessions, the participants have reported feeling relaxed, free from pressing thoughts about external factors, letting free and to go with the flow to relish the present moment. These perceived outcomes resonate with the concept central to DMP where it is argued that the active engagement in the process generates vitality and joy due to dance as an art form and also because of the physiological changes as an exercise [41,65].

Apart from the common findings between the Indian [42,43] and the current study as describe above, there were also important differences between them. A striking difference is the ease in which unpleasant emotions and darker aspects of self were revealed in the Indian study in contrast with the UK groups. Although people use combination of coping strategies, one of the emotion-focused coping strategies predominantly used by most of the participants in the Indian sample was releasing pent-up emotions as early as in the second session. Besides, social coping was also noticed within the UK group with people avoiding opening up emotionally, using the session as escape, and compartmentalizing thoughts and emotions. In addition, the participants of the current study reported problem-focused strategies such as taking control, planning ahead and appraisal-focused strategies such as altering plans and adapting to the situation as preferred choices. Charles et al. [66] argued that it is highly unlikely that people with more personal and environmental resources would prefer avoidance coping. On the contrary, the stress levels of participants in India were much higher and environmental resources were lesser. So, one of the possible explanations for the differences could be the collectivist and individualistic cultural differences. The British participants coming from a less collectivist culture, might have found it more difficult to feel part of the group at first and trust the group members from the very beginning; while the Indian group had vocabulary and felt comfortable to express unpleasant emotions in a group context. Another major difference was the ease with which the Indian participants engaged with the movement and creative components of the DMP sessions. This could be possibly because of the familiarity in using their bodies creative expressions and also engage with emotional and symbolic content [67].

DMP relies heavily on expressing difficult emotions using symbolism and metaphor, transforming unhelpful emotions into healthy actions and finding resolutions to one’s unresolved issues [68,69]. Concrete thinking, wishful thinking, intellectualising defences, and distancing from deeper emotions were not supportive to engage in DMP and this could explain high attrition rates. As mentioned in the results, one possible rationalisation was that it is children who require intervention and as a caregiver, skill set, strategies and techniques are more important. This attitude can also be found in the literature as well where the demand and number of interventions for parenting skill development are more than wellbeing and self-care programmes [32]. However, other possible explanations for challenges to engage in DMP are that as there were several distractions within the school environment to contain one of the groups and because of which the participants might have reacted defensively and not felt safe to express or needed more time to feel safe [70]. With regards to teachers, their professional relationships and dynamics could have held them back from expressing unpleasant feelings and emotions [24]. It is, therefore, worth considering this dual relationship as a sensitive factor to build the group and offer a safe therapeutic space.

### Limitations and future recommendations

The quality of the data could have been influenced by poor acoustic, video recording angles of the camera and room dimensions. Hence use of multiple cameras and cameras with motion capture functions can further the quality of movement related investigation. In addition, English not being the native language of the researcher could have impacted the data collection and analysis. Reflections from the participants were clarified with the participants by paraphrasing it to them during the sessions and later transcripts were verified by a native English speaker and researcher. However, due to time restrains and potential emotional burden it was not possible to get the participants to comment or review the transcript or conduct follow up interviews.

Although there were some similarities across four groups, data saturation was not noticed in the research process as new information was still being shared in different sessions [71]. In addition, the heterogeneity in the sample makes the topic fertile for further in-depth exploration of the nuances. Inconsistent attendance of the caregivers may have impacted their experience and the quality of the data. One of the reasons could have been because of the separate sessions for caregivers and children. The concept of child and caregiver seen separately by the same therapist is a well-accepted model in verbal child psychotherapy [72,73]. Since, it is novel to DMP, further thoughts around enhancing caregivers’ engagement are warranted. Blauth [36] who conducted parental counselling sessions while providing music therapy to children reported good attendance and positive results. The differences are verbal v/s nonverbal or creative and individual v/s group which appears to have impacted this study drastically. Parsons and Dubrow-Marshall [59] in their article, discuss about the polarities of themes stressing on the intrinsic undercurrents in delivering creative, unconventional, and embodied forms of therapies within a school environment. The authors suggested that although, there are no quick solutions to the constraints, normative inhibitions and expectations of the participants and wider school structures and community mind-set; taking cognizance of the tension between helpful and unhelpful factors might be valuable for dance movement psychotherapists in setting themselves. Regardless of being aware of the school dynamics and taking rigorous actions, it was still hard to consistently establish a therapeutically safe environment within the school settings. Future research could involve different stakeholders and lived experience consultants to discuss the practical strategies to manage the glitches pertaining to SEN settings. In addition, a collective effort the therapists’ community to sensitize and create awareness about the work of arts therapies within the school management, and amongst staff and parents could help in establishing sustainable interventions.

## Conclusions

The study offers multiple perspectives and presents the experiences shared by the participants after attending DMP sessions in SEN school settings. In this MRC framework informed study, caregivers offered a positive review and acknowledged that a strength-based DMP intervention was helpful. Participants appreciated the creative and expressive nature of the intervention to promote their emotional and social wellbeing. The study also identified many helpful and unhelpful factors that influenced the process and outcomes of DMP with caregivers of children on the autism spectrum. The findings of this study merit further exploration through a larger study. However, it is highly recommended that barriers to caregiver engagement identified in this study need to be addressed prior to the inception of a larger study by involving lived experience consultants and other stakeholders within the research team. We propose a collective approach to sensitize and create awareness about the work of arts therapists within the school management, and amongst staff and parents to establish sustainable and accessible support and care for caregivers of children on the autism spectrum.

## Supporting information

S1 Table Descriptive statistics of the participants background characteristics in the DMP intervention and standard care groups, separated by teacher/parent status. (DOCX)

S2 Table Qualitative themes codebook for caregivers. (DOCX)

## References

[1] Zeidan J, Fombonne E, Scorah J, Ibrahim A, Durkin MS, Saxena S, et al. Global prevalence of autism: A systematic review update. Autism Res. 2022 May;15(5):778–90. doi: 10.1002/aur.2696 35238171 PMC9310578

[2] World Health Organization. Autism [Internet]. 2018 [cited 2022 Sep 1]. Available from: https://www.who.int/news-room/fact-sheets/detail/autism-spectrum-disorders.

[3] World Health Organization. Comprehensive and coordinated efforts for the management of autism spectrum disorders. World Health Organization, Executive Board 133rd Session Provisional Agenda Item. 2013 Apr 8;6:14.

[4] Yorke I, White P, Weston A, Rafla M, Charman T, Simonoff E. The Association Between Emotional and Behavioral Problems in Children with Autism Spectrum Disorder and Psychological Distress in Their Parents: A Systematic Review and Meta-analysis. J Autism Dev Disord. 2018 Oct;48(10):3393–415. doi: 10.1007/s10803-018-3605-y 29777471 PMC6153902

[5] Gray DE. ‘Everybody just freezes. Everybody is just embarrassed’: felt and enacted stigma among parents of children with high functioning autism. Sociology of Health & Illness. 2002;24(6):734–49.

[6] Peters-Scheffer N, Didden R, Korzilius H, Matson J. Cost comparison of early intensive behavioral intervention and treatment as usual for children with autism spectrum disorder in The Netherlands. Res Dev Disabil. 2012 Dec;33(6):1763–72. doi: 10.1016/j.ridd.2012.04.006 22705454

[7] Giovagnoli G, Postorino V, Fatta LM, Sanges V, De Peppo L, Vassena L, et al. Behavioral and emotional profile and parental stress in preschool children with autism spectrum disorder. Res Dev Disabil. 2015 Nov;45–46:411–21. doi: 10.1016/j.ridd.2015.08.006 26318505

[8] Barroso NE, Mendez L, Graziano PA, Bagner DM. Parenting Stress through the Lens of Different Clinical Groups: a Systematic Review & Meta-Analysis. J Abnorm Child Psychol. 2018 Apr;46(3):449–61. 28555335 10.1007/s10802-017-0313-6 PMC5725271

[9] Ni’matuzahroh, Ningrum V, Widayat, Dyah Artaria M, Suen MW. The COVID-19 pandemic and healthcare workers psychological well-being: a cross-sectional survey in Indonesia. Nursing Open. 2021 Nov;8(6):3212–21. doi: 10.1002/nop2.1034 34427392 PMC8510726

[10] Porter N, Loveland KA, Saroukhani S, Posey Y, Morimoto K, Rahbar MH. Severity of Child Autistic Symptoms and Parenting Stress in Mothers of Children with Autism Spectrum Disorder in Japan and USA: Cross-Cultural Differences. Autism Res Treat. 2022;2022:7089053. doi: 10.1155/2022/7089053 35864923 PMC9296302

[11] Senthil D M. Relationship between family interaction, family burden and quality of life among caregivers of patients with epilepsy. International Journal of Research–Granthaalayah. 2017 Aug 22;4(4):108–14.

[12] Lancastle D, Hill J, Faulkner S, Cousins AL. “The stress can be unbearable, but the good times are like finding gold”: A phase one modelling survey to inform the development of a self-help positive reappraisal coping intervention for caregivers of those with autism spectrum disorder. PLOS ONE. 2022 Mar 3;17(3):e0264837. doi: 10.1371/journal.pone.0264837 35239745 PMC8893638

[13] Brezis RS. Memory integration in the autobiographical narratives of individuals with autism. Frontiers in Human Neuroscience [Internet]. 2015 [cited 2022 Sep 1];9. Available from: https://www.frontiersin.org/articles/10.3389/fnhum.2015.00076. 25741270 10.3389/fnhum.2015.00076 PMC4327287

[14] De Stasio S, Fiorilli C, Benevene P, Uusitalo-Malmivaara L, Di Chiacchio C. Burnout in special needs teachers at kindergarten and primary school: investigating the role of personal resources and work wellbeing. Psychology in the Schools. 2017 May;54(5):472–86.

[15] Carmichael C, Callingham R, Watt H. Classroom motivational environment influences on emotional and cognitive dimensions of student interest in mathematics. ZDM. 2017 Jan 18;49.

[16] DfE. Educational excellence everywhere [Internet]. GOV.UK. 2016 [cited 2022 Sep 1]. Available from: https://www.gov.uk/government/publications/educational-excellence-everywhere.

[17] Ault S, Breitenstein SM, Tucker S, Havercamp SM, Ford JL. Caregivers of Children with Autism Spectrum Disorder in Rural Areas: A Literature Review of Mental Health and Social Support. J Pediatr Nurs. 2021 Nov 1;61:229–39. doi: 10.1016/j.pedn.2021.06.009 34153794

[18] Parkes A. Parenting stress and parent support among mothers with high and low education. Journal of Family Psychology. 2015 0720;29(6):907. doi: 10.1037/fam0000129 26192130 PMC4671474

[19] Shepherd D, Goedeke S, Landon J, Meads J. The Types and Functions of Social Supports Used by Parents Caring for a Child With Autism Spectrum Disorder. J Autism Dev Disord. 2020 Apr 1;50(4):1337–52. doi: 10.1007/s10803-019-04359-5 31919701

[20] Lachman ME, Weaver SL. The sense of control as a moderator of social class differences in health and well-being. J Pers Soc Psychol. 1998 Mar;74(3):763–73. doi: 10.1037//0022-3514.74.3.763 9523418

[21] Brunsting NC, Sreckovic MA, Lane KL. Special education teacher burnout: A Synthesis of Research from 1979 to 2013. Education & Treatment of Children. 2014;37(4):681–712.

[22] Brown CP, Lan YC. A qualitative metasynthesis comparing U.S. teachers’ conceptions of school readiness prior to and after the implementation of NCLB. Teaching and Teacher Education. 2015 Jan 1;45:1–13.

[23] Glazzard J, Rose A. The impact of teacher well-being and mental health on pupil progress in primary schools. Journal of Public Mental Health. 2019 Jan 1;19(4):349–57.

[24] Van Droogenbroeck F, Spruyt B, Vanroelen C. Burnout among senior teachers: Investigating the role of workload and interpersonal relationships at work. Teaching and Teacher Education. 2014;43:99–109.

[25] Pillay H, Goddard R, Wilss L. Well-Being, Burnout and Competence: Implications for Teachers. Australian Journal of Teacher Education [Internet]. 2005 Nov 1;30(2). Available from: https://ro.ecu.edu.au/ajte/vol30/iss2/3.

[26] Altiere MJ, von Kluge S. Family Functioning and Coping Behaviors in Parents of Children with Autism. J Child Fam Stud. 2009 Feb;18(1):83–92.

[27] Greeff AP, van der Walt KJ. Resilience in Families with an Autistic Child. Education and Training in Autism and Developmental Disabilities. 2010 Sep;45(3):347–55.

[28] De Stasio S, Fiorilli C, Di Chiacchio C. Effects of verbal ability and fluid intelligence on children’s emotion understanding. Int J Psychol. 2014 Oct;49(5):409–14. doi: 10.1002/ijop.12032 25178964

[29] Flores MA, Day C. Contexts which shape and reshape new teachers’ identities: A multi-perspective study. Teaching and Teacher Education. 2006 Feb;22(2):219–32.

[30] Day C, Leitch R. Teachers’ and teacher educators’ lives: the role of emotion. Teaching and Teacher Education. 2001 May 1;17(4):403–15.

[31] Rezendes DL, Scarpa A. Associations between Parental Anxiety/Depression and Child Behavior Problems Related to Autism Spectrum Disorders: The Roles of Parenting Stress and Parenting Self-Efficacy. Autism Research and Treatment. 2011 Dec 13;2011:e395190. doi: 10.1155/2011/395190 22937246 PMC3420762

[32] Fewster DL, Govender P, Uys CJ. Quality of life interventions for primary caregivers of children with autism spectrum disorder: a scoping review. J Child Adolesc Ment Health. 2019 Sep;31(2):139–59. doi: 10.2989/17280583.2019.1659146 31570089

[33] McCubbin HI, McCubbin MA, Thompson AI, Thompson EA. Resiliency in ethnic families: A conceptual model for predicting family adjustment and adaptation. In: Resiliency in Native American and immigrant families. Thousand Oaks, CA, US: Sage Publications, Inc; 1998. p. 3–48. (Resiliency in families series, Vol. 2.).

[34] Davis H. & Day C. (2010). Working in Partnership: The Family Partnership Model. London: Pearson. 2010.

[35] Bernal G, Jiménez-Chafey MI, Domenech Rodríguez MM. Cultural adaptation of treatments: A resource for considering culture in evidence-based practice. Professional Psychology: Research and Practice. 2009 Aug;40(4):361–8.

[36] Blauth L. Music therapy and parent counselling to enhance resilience in young children with autism spectrum disorder: a mixed methods study [Internet] [doctoral]. Anglia Ruskin University; 2019 [cited 2022 Sep 1]. Available from: https://arro.anglia.ac.uk/id/eprint/704640/.

[37] Pasiali V. Family-based Music Therapy: Fostering Child Resilience and Promoting Parental Self-efficacy Through Shared Musical Experiences. Michigan State University. Department of Music Education; 2010. 544 p.

[38] Thompson G. Making a connection: randomised controlled trial of family centred music therapy for young children with autism spectrum disorder [Internet]. University of Melbourne; 2012 [cited 2022 Sep 1]. Available from: https://findanexpert.unimelb.edu.au/scholarlywork/1471133-making-a-connection—randomised-controlled-trial-of-family-centred-music-therapy-for-young-children-with-autism-spectrum-disorder.

[39] Shim M, Johnson RB, Gasson S, Goodill S, Jermyn R, Bradt J. A model of dance/movement therapy for resilience-building in people living with chronic pain. European Journal of Integrative Medicine. 2017 Jan 1;9:27–40.

[40] Karkou V, Meekums B. Dance movement therapy for dementia. Cochrane Database Syst Rev. 2017 Feb 3;2017(2):CD011022. doi: 10.1002/14651858.CD011022.pub2 28155990 PMC6464250

[41] Karkou V, Aithal S, Zubala A, Meekums B. Effectiveness of Dance Movement Therapy in the Treatment of Adults With Depression: A Systematic Review With Meta-Analyses. Frontiers in Psychology [Internet]. 2019 [cited 2022 Sep 1];10. Available from: https://www.frontiersin.org/articles/10.3389/fpsyg.2019.00936. 31130889 10.3389/fpsyg.2019.00936 PMC6509172

[42] Aithal S, Karkou V, Kuppusamy G, Mariswamy P. Backing the backbones—A feasibility study on the effectiveness of dance movement psychotherapy on parenting stress in caregivers of children with Autism Spectrum Disorder. The Arts in Psychotherapy. 2019 Jul 1;64:69–76.

[43] Aithal S, Karkou V, Kuppusamy G. Resilience enhancement in parents of children with an autism spectrum disorder through dance movement psychotherapy. The Arts in Psychotherapy. 2020 Nov 1;71:101708.

[44] Skivington K, Matthews L, Simpson SA, Craig P, Baird J, Blazeby JM, et al. A new framework for developing and evaluating complex interventions: update of Medical Research Council guidance. bmj. 2021 Sep 30;374. doi: 10.1136/bmj.n2061 34593508 PMC8482308

[45] Heidegger M. The Basic Problems of Phenomenology, Revised Edition. Revised edition. Bloomington (Ind.): Indiana University Press; 1988. 432 p.

[46] Hoffmann TC, Glasziou PP, Boutron I, Milne R, Perera R, Moher D, et al. Better reporting of interventions: template for intervention description and replication (TIDieR) checklist and guide. BMJ. 2014 Mar 7;348:g1687. doi: 10.1136/bmj.g1687 24609605

[47] Aithal S. Dance movement psychotherapy for the wellbeing of children on the autism spectrum and their caregivers: a mixed-methods study [Internet] [Ph.D.]. Edge Hill University; 2020 [cited 2022 Sep 2]. Available from: https://research.edgehill.ac.uk/en/studentTheses/ceb90daf-54d2-4797-9208-1e071f828af0.

[48] Parsons A, Omylinska-Thurston J, Karkou V, Harlow J, Haslam S, Hobson J, et al. Arts for the blues–a new creative psychological therapy for depression. British Journal of Guidance & Counselling. 2020 Jan 2;48(1):5–20.

[49] Johnson B, Christensen L. Educational Research: Quantitative, Qualitative, and Mixed Approaches. SAGE; 2010. 649 p.

[50] Asan O, Montague E. Using video-based observation research methods in primary care health encounters to evaluate complex interactions. jhi. 2014 Aug 14;21(4):161–70. doi: 10.14236/jhi.v21i4.72 25479346 PMC4350928

[51] Hervey LW. Artistic Inquiry in Dance/Movement Therapy: Creative Research Alternatives. Springfield, Ill: Charles C Thomas Pub Ltd; 2000. 158 p.

[52] Braun V, Clarke V. Using thematic analysis in psychology. Qualitative Research in Psychology. 2006 Jan 1;3(2):77–101.

[53] Riessman CK. Narrative analysis. Thousand Oaks, CA, US: Sage Publications, Inc; 1993. vii, 79 p. (Narrative analysis).

[54] Lapadat JC, Lindsay AC. Transcription in Research and Practice: From Standardization of Technique to Interpretive Positionings. Qualitative Inquiry. 1999 Mar;5(1):64–86.

[55] QSR. Quality and Standards Review (England) [Internet]. 2017 [cited 2022 Sep 1]. Available from: https://www.qaa.ac.uk/reviewing-higher-education/types-of-review/quality-and-standards-review.

[56] Lincoln YS, Guba E. Naturalistic Inquiry. 1st edition. Beverly Hills, Calif: SAGE Publications, Inc; 1985. 416 p.

[57] Najmi A, Raza SA, Qazi W. Does statistics anxiety affect students’ performance in higher education? The role of students’ commitment, self-concept and adaptability. International Journal of Management in Education. 2018 Jan 1;12:95.

[58] Koch SC, Riege RFF, Tisborn K, Biondo J, Martin L, Beelmann A. Effects of Dance Movement Therapy and Dance on Health-Related Psychological Outcomes. A Meta-Analysis Update. Front Psychol. 2019 Aug 20;10:1806. doi: 10.3389/fpsyg.2019.01806 31481910 PMC6710484

[59] Parsons AS, Dubrow-Marshall L. ‘Putting themselves out there’ into the unknown: Dance movement psychotherapy as perceived by five educators and three pupils. Body, Movement and Dance in Psychotherapy. 2018 Oct 2;13(4):251–67.

[60] Schacter D. Psychology: European Edition. European ed edition. Basingstoke: Palgrave; 2011. 784 p.

[61] Miranda JJ, Barrientos-Gutiérrez T, Corvalan C, Hyder AA, Lazo-Porras M, Oni T, et al. Understanding the rise of cardiometabolic diseases in low- and middle-income countries. Nat Med. 2019 Nov;25(11):1667–79. doi: 10.1038/s41591-019-0644-7 31700182

[62] Tugade MM, Fredrickson BL. Resilient Individuals Use Positive Emotions to Bounce Back From Negative Emotional Experiences. Journal of Personality and Social Psychology. 2004;86(2):320–33. doi: 10.1037/0022-3514.86.2.320 14769087 PMC3132556

[63] Fredrickson BL, Joiner T. Positive emotions trigger upward spirals toward emotional well-being. Psychol Sci. 2002 Mar;13(2):172–5. doi: 10.1111/1467-9280.00431 11934003

[64] Seligman MEP, Rashid T, Parks AC. Positive psychotherapy. American Psychologist. 2006;61(8):774–88. doi: 10.1037/0003-066X.61.8.774 17115810

[65] Jola C, Calmeiro L. The dancing queen: Explanatory mechanisms of the ‘feel-good-effect’ in dance. In: The Oxford Handbook of Dance and Wellbeing. 2017.

[66] Charles RI, Charles R, Lester FK, O’Daffer PG. How to Evaluate Progress in Problem Solving. National Council of Teachers of Mathematics; 1987. 104 p.

[67] Kim BSK, Atkinson DR, Yang PH. The Asian Values Scale: Development, factor analysis, validation, and reliability. Journal of Counseling Psychology. 1999;46(3):342–52.

[68] Meekums B. Dance Movement Therapy: A Creative Psychotherapeutic Approach [Internet]. London; 2002 [cited 2022 Sep 2]. Available from: https://sk.sagepub.com/books/dance-movement-therapy.

[69] Karkou V, Sanderson P. Arts Therapies: A Research-based Map of the Field. Elsevier Health Sciences; 2006. 316 p.

[70] Finlay L. Relational integrative psychotherapy: Engaging process and theory in practice. Wiley-Blackwell; 2016. xiii, 260 p. (Relational integrative psychotherapy: Engaging process and theory in practice).

[71] Saunders B, Sim J, Kingstone T, Baker S, Waterfield J, Bartlam B, et al. Saturation in qualitative research: exploring its conceptualization and operationalization. Qual Quant. 2018;52(4):1893–907. doi: 10.1007/s11135-017-0574-8 29937585 PMC5993836

[72] Chazan SE. Simultaneous Treatment of Parent and Child: Second Edition. 2nd edition. London; New York: Jessica Kingsley Publishers; 2003. 240 p.

[73] Nilsson M. To Be the Sole Therapist: Children and Parents in Simultaneous Psychotherapy. Journal of Infant, Child, and Adolescent Psychotherapy. 2006 Apr 1;5(2):206–25.

