# Supplementary material for: Supporting the wellbeing of caregivers of children on the autism spectrum: A qualitative report on experiences of attending group dance movement psychotherapy
Source: PLoS One. 2023 Aug 4;18(8):e0288626. doi: 10.1371/journal.pone.0288626 (PMC10403118; doi:10.1371/journal.pone.0288626)
Supplement: S1 Table — (DOCX) [file pone.0288626.s001.docx]

**Table 1. Descriptive statistics of the participants background characteristics in the DMP intervention and standard care groups, separated by teacher/parent status.**

| **Variables** | | **DMP Intervention** | |
| --- | --- | --- | --- |
|  |  | **Teachers** | **Parents** |
| Centre | Location 1 (n) | 4 | 5 |
|  | Location 2 (n) | 5 | 6 |
| Age (mean) and range | | 39.4  (32-56) | 43.6  (36-51) |
| Gender (n) F- Female M-Male | | 9 F | 9F, 2M |
| Ethnicity, (n) | White | 9 | 9 |
|  | Black | - | 2 |
|  | Asian and others | - | - |
| Marital Status (n) | Single parent | 2 | 6 |
|  | Married | 4 | 3 |
|  | Cohabiting | 3 | 2 |
| Number of children (median) | | 2 | 2 |
| Number of children attending SEN (median) | | - | 2 |
| Number of participants with at least 70% attendance | | 6 | 4 |
