# Supplementary material for: Supporting the wellbeing of caregivers of children on the autism spectrum: A qualitative report on experiences of attending group dance movement psychotherapy
Source: PLoS One. 2023 Aug 4;18(8):e0288626. doi: 10.1371/journal.pone.0288626 (PMC10403118; doi:10.1371/journal.pone.0288626)
Supplement: S2 Table — (DOCX) [file pone.0288626.s002.docx]

**Table 2. Qualitative themes codebook for caregivers**

| Sl.no | Themes | Frequency of occurrence | Session Numbers (1-5) |
| --- | --- | --- | --- |
| 1 | **Beholding within and around**   - *1.1Enhancing awareness and alertness* - *Unlocking the unfamiliar* | 21 | 1, 3 and 5 |
| 2 | **Reflecting and reinforcing strengths**   - *Embracing Positivity* - *Recalling fun moments with children* | 18 | 2 and 3 |
| 3 | **Exchanging views**   - *Structuring and compartmentalising* - *Sense of control* - *Escape* - *Confronting the challenges* - *Planning ahead* - *Acceptance* | 19 | 3 and 4 |
| 4 | **Looking back and carrying forward**   - *Unprocessed baggage* - *Creative action plan* | 16 | 1, 4 and 5 |
| 5 | **Core benefits**   - *Relaxation* - *Self-expression* - *Grounding and relishing the present* - *Letting go* | 23 | 1, 4 and 5 |
| 6 | **Challenges to engage in DMP**   - *Contemplation about the approach* - *Readiness to trust and be playful* - *Access to emotional and symbolic content* - *Therapeutically safe environment* | 14 | 1, 2, 3 and 4 |
